# Tracking the return of *Aedes aegypti* to Brazil, the major vector of the dengue, chikungunya and Zika viruses

**DOI:** 10.1371/journal.pntd.0005653

**Published:** 2017-07-25

**Authors:** Panayiota Kotsakiozi, Andrea Gloria-Soria, Adalgisa Caccone, Benjamin Evans, Renata Schama, Ademir Jesus Martins, Jeffrey R. Powell

**Affiliations:** 1 Department of Ecology and Evolutionary Biology, Yale University, New Haven, Connecticut, United States of America; 2 Laboratório de Biologia Computacional e Sistemas, IOC–Fiocruz, Rio de Janeiro, Brazil; 3 Laboratório de Fisiologia e Controle de Artrópodes Vetores, IOC-FIOCRUZ, Rio de Janeiro, Brazil; University of Texas Medical Branch, UNITED STATES

## Abstract

**Background:**

*Aedes aegypti*, commonly known as “the yellow fever mosquito”, is of great medical concern today primarily as the major vector of dengue, chikungunya and Zika viruses, although yellow fever remains a serious health concern in some regions. The history of *Ae*. *aegypti* in Brazil is of particular interest because the country was subjected to a well-documented eradication program during 1940s-1950s. After cessation of the campaign, the mosquito quickly re-established in the early 1970s with several dengue outbreaks reported during the last 30 years. Brazil can be considered the country suffering the most from the yellow fever mosquito, given the high number of dengue, chikungunya and Zika cases reported in the country, after having once been declared “free of *Ae*. *aegypti*”.

**Methodology/Principal findings:**

We used 12 microsatellite markers to infer the genetic structure of Brazilian *Ae*. *aegypti* populations, genetic variability, genetic affinities with neighboring geographic areas, and the timing of their arrival and spread. This enabled us to reconstruct their recent history and evaluate whether the reappearance in Brazil was the result of re-invasion from neighboring non-eradicated areas or re-emergence from local refugia surviving the eradication program. Our results indicate a genetic break separating the northern and southern Brazilian *Ae*. *aegypti* populations, with further genetic differentiation within each cluster, especially in southern Brazil.

**Conclusions/Significance:**

Based on our results, re-invasions from non-eradicated regions are the most likely scenario for the reappearance of *Ae*. *aegypti* in Brazil. While populations in the northern cluster are likely to have descended from Venezuela populations as early as the 1970s, southern populations seem to have derived more recently from northern Brazilian areas. Possible entry points are also revealed within both southern and northern clusters that could inform strategies to control and monitor this important arbovirus vector.

## Author summary

*Aedes aegypti* (“yellow fever mosquito”) is of great medical concern because it is the primary vector of important viruses causing dengue fever, chikungunya and Zika, as well as increasingly transmitting yellow fever once again. Due to the Zika outbreak, which started in Brazil in 2015 and rapidly spread with million cases reported in Central/South America and the Caribbean, this mosquito has attained special notoriety. Brazil is by far the most affected country not only by Zika, but also by dengue and chikungunya. Interestingly, in 1950s Brazil had been declared “free of *Ae*. *aegypti*” after a well-documented eradication program. However, during the last 45 years *Ae*. *aegypti* reappeared in Brazil causing several dengue outbreaks, with millions of reported cases. Here, we used genetic data to study the reappearance of *Ae*. *aegypti* in Brazil after the putative eradication event. Our results support re-invasion; mosquitoes from the non-eradicated areas in Venezuela invaded North Brazil, later expanding their distribution southwards. We also identify specific locations in Brazil, as possible entry points. This knowledge can inform strategies to control the spread of the vector and of the diseases it transmits.

## Introduction

*Aedes aegypti* is one of the most successful worldwide invaders, spreading from its native Africa to most tropical and subtropical regions of the world [1,2] and it is the primary vector of dengue fever, chikungunya and Zika viruses. There is no vaccine for chikungunya and Zika and it is still unclear whether there is an effective vaccine for dengue, so control of *Ae*. *aegypti* remains the major target for disease control. The species’ highly anthropophilic behavior, its ability to lay desiccation-resistant eggs, its high passive dispersal, and its tendency to develop pesticide-resistance [3,4], have made the control of this vector extremely difficult. Since *Ae*. *aegypti*’s arrival in the Americas, shortly after Europeans in the 16^th^ Century, many dengue outbreaks have been recorded, including several in Brazil and the Caribbean [5]. Brazil together with other South American and Caribbean countries were subjected to an intense eradication program during 1940s-1950s and in the late 1950s [6] were declared *Ae*. *aegypti* free. However, after the relaxation of the eradication campaigns, *Ae*. *aegypti* reappeared in some of these regions. This could have happened either through re-invasion from non-eradicated regions, such as Venezuela, USA, and some Caribbean islands [6], or recrudescence from relict populations, or a combination of both.

The study of the genetic structure and the identification of likely source populations or isolated Brazilian populations that may be efficiently targeted for control can contribute to develop better strategies to control the spread of this vector and of the diseases that it transmits. It can also provide insights on the roles of environmental and human induced factors that can contribute to vector or disease spread.

**Fig 1:**
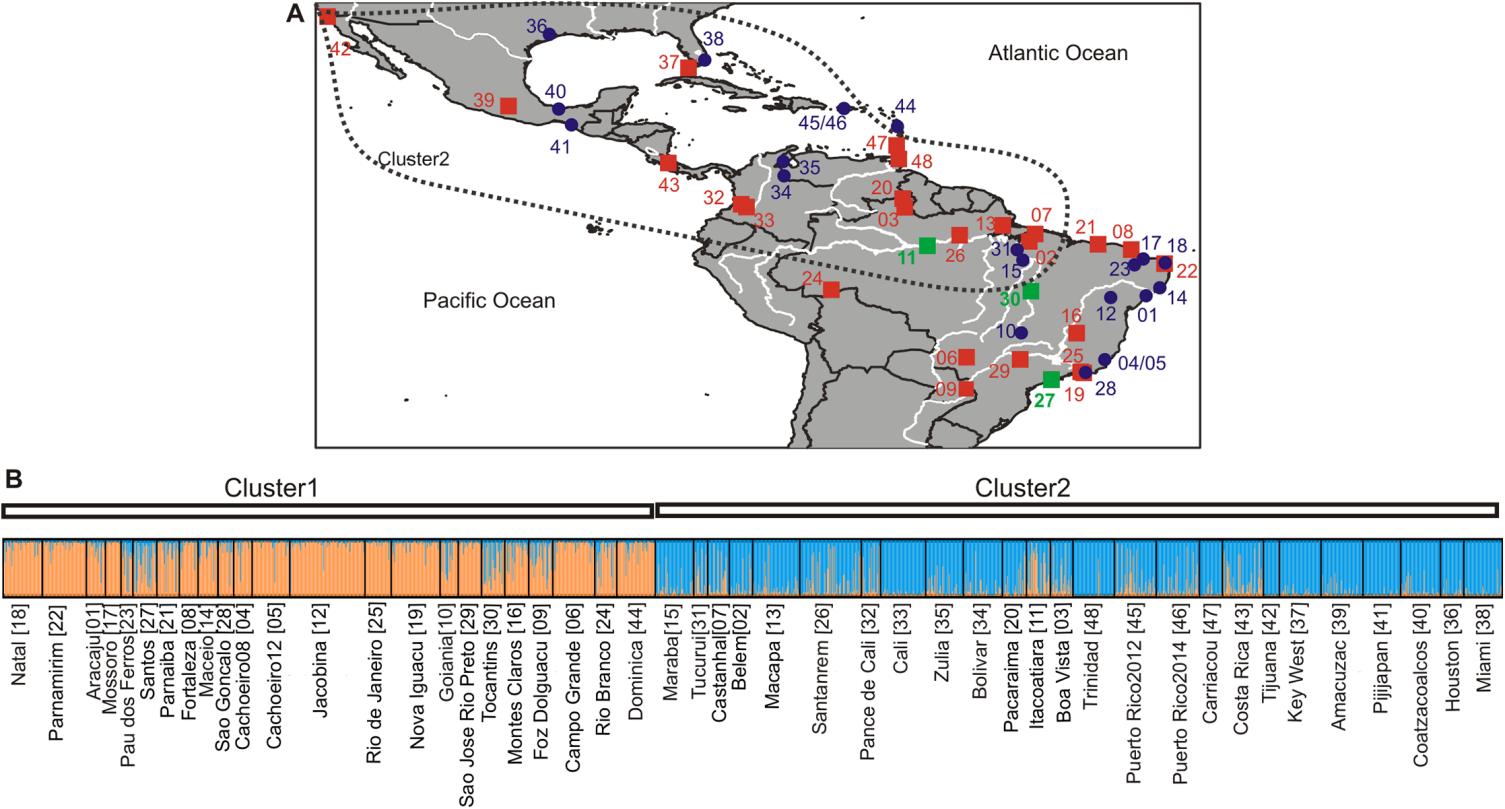
Collection sites and preliminary STRUCTURE analysis. Collection sites for all the *Ae*. *aegypti* populations used in this study (A). Populations studied previously [7] are indicated as blue dots whereas the new collections as red squares. Three newly added populations that exhibited signs of admixed ancestry in STRUCTURE are indicated with green squares. The area included in the dashed line shows the northern genetic Cluster identified by Monteiro et al. [7] and confirmed in this study as indicated by STRUCTURE (B). Code numbers are as in S1 Table. STRUCTURE bar plot for all *Ae*. *aegypti* populations used in this study (B). Population names are reported on its X axis, with numbers as in Fig 1A and S1 Table. The Y axis reports the probability of each individual (Q-value) assigned to one of the two genetic groups identified by STRUCTURE, which are represented by different colors (blue and orange). Each bar represents an individual. Individuals with 100% assignment to one group are identified by a single color. Individuals with mixed ancestry are represented by bars with different percentages of the two colors. The thick black lines within the plots indicate population limits.

A few studies have provided insights on levels of genetic diversity in Brazilian populations. Microsatellites and mitochondrial DNA (mtDNA) revealed moderate [7] to high [8] levels of genetic differentiation between populations, limited gene flow and dispersal capability, especially in urban areas [9]. At the country level, mtDNA [10], insecticide resistant alleles data [11] and microsatellites [7] have shown the existence of two genetically distinct groups in Brazil. According to Monteiro et al. [7] *Ae*. *aegypti* from the east, southern and central Brazil are genetically close to populations from the island of Dominica in the Caribbean, whereas the populations from northern Brazil have genetic affinities primarily with northern South American countries (Fig 1). This suggest that the reappearance of *Ae*. *aegypti* in Brazil was likely due to two independent re-invasions from non-eradicated neighboring areas, possibly Dominica in the Caribbean and northern South America.

In this study we revisit the analysis of *Ae*. *aegypti* genetic diversity in Brazil by screening for genetic diversity at 12 microsatellite loci in 48 Brazilian and non-Brazilian populations, a much denser spatial sampling than in previous studies. We use these data to study the patterns of genetic differentiation among populations of *Ae*. *aegypti* in Brazil at a fine geographic scale, to describe their genetic affinities with populations from neighboring regions from both South and North/Central America and the Caribbean, and to reconstruct the history of the re-appearance of *Ae*. *aegypti* in Brazil.

## Methods

### Mosquito collections

*Aedes aegypti* samples were collected from 46 locations (2 locations were sampled twice, so 48 samples in total) in America and Caribbean (Fig 1A and S1 Table). Samples were either adults from the field or eggs that were collected in multiple ovitraps per location to minimize sampling siblings. Brazilian samples were all collected in ovitraps, following the Brazilian National Network for Monitoring Insecticide Resistance in *Ae*. *aegypti* (Morenaa Network) recommended procedures [12]. Samples from generation F0 to F2 (39 out of 48 were F0; S1 Table) were preserved in 70–100% ethanol or dry at -80°C until DNA extraction. Thirty-one samples from Brazil and 17 from outside Brazil (1,875 individual *Ae*. *aegypti* mosquitoes in total) were used in the present study (for details see S1 Table). Twelve out of the 31 Brazilian populations and 8 non Brazilian had been studied previously [7] and their data are included in this study (Fig 1).

### DNA extraction and microsatellite genotyping

Total DNA was extracted using the DNeasy Blood and Tissue kit (Qiagen) according to the manufacturer instructions, with an additional RNAse A (Qiagen) step. DNA samples were stored at -20^°^C until further analysis. Individual mosquitoes were genotyped as described in Brown et al. [13] for twelve previously published [2,13,14] microsatellite loci [A1, B2, B3, A9 (tri-nucleotide repeats), and AC2, CT2, AG2, AC4, AC1, AC5, AG1, and AG4 (di-nucleotide repeats)]. Microsatellite alleles were scored using GENEMAPPER version 4.0 (Applied Biosystems). Raw allele frequencies are available at VectorBase.org, Project ID VBP0000176 and are also available as Supporting Information (S1 Appendix).

### Genetic diversity and differentiation

All microsatellite loci were analyzed for within-population deviations from Hardy-Weinberg equilibrium (HWE) using the exact HWE test in GENEPOP v.4.5.1 [15,16]. The same software was used for the estimations of the Linkage disequilibrium (LD) among all pairs of loci. Both HWE and LD tests were run with 10,000 dememorizations, 1,000 batches and 10,000 iterations per batch. Bonferroni corrections were applied to the resulting matrices of both HWE and LD. Allele numbers, allelic frequencies, and average observed (Ho) and expected (He) heterozygosities were estimated using GenAlEx [17]. In order to test if the mean observed heterozygosity is significantly lower than mean expected heterozygosity for each one of the studied populations, we performed paired t tests (the homogeneity of variances tested with Barlett's test). GeneAlex was also used for the Analysis of Molecular Variance (AMOVA). Allelic richness (AR) and private allelic richness (Np) were calculated in HPRARE [18,19]. One-way ANOVA was used to test for significant differences on Ho and allelic richness levels between different groups of populations (see results). The probability of null allele occurrence was tested for each population and each locus separately using MICROCHECKER v2.2.3 [20]. The pairwise genetic distances (Fst) and their significance were calculated in Arlequin v3.5.1.2 [21], using 1,000 permutations. To estimate the extent of bias in the Fst estimations because of the presence of null alleles in our dataset, the FreeNA software [22] was used with 1,000 bootstraps replications.

### Population structure

Geographic population structure was evaluated using the Bayesian clustering method implemented in the software STRUCTURE v.2.3 [23], which identifies genetic clusters and assigns individuals to these clusters with no *a priori* information of sampling location. We conducted different runs using different datasets (see results).

For each dataset the most likely number of clusters (K), was determined by conducting 10 independent runs for each K = 1 to the maximum number of populations included in the analysis. Each run assumed an admixture model and independent allele frequencies (lambda set to one), using a burn-in value of 100,000 iterations followed by 500,000 repetitions. The optimal number of K clusters was determined using the Delta K method of Evanno et al. [24], using the online version of STRUCTURE HARVESTER v.0.6.94 [25]. The program CLUMPP v1.1.2 [26] was used to summarize the results from the 10 independent STRUCTURE runs and provide the Q matrices based on which a population or an individual can be assigned to a specific cluster. The results were plotted using DISTRUCT v.1.1 [27].

To complement the Bayesian analysis, we also performed a Discriminant Analysis of Principal Components (DAPC), using the “find.clusters” option of ADEGENET package [28] in R v.3.1.3 (R Core Team 2015) so in this case, individuals assigned to DAPC-defined clusters.

### Isolation by distance (IBD)

Mantel tests to assess the significance of correlation between geographic (Euclidean distance as estimated in R based on the localities’ coordinates; S1 Table) and genetic (Fst) distance matrices were performed on different groups, based on the results from the genetic structure analyses (see results). The Mantel tests were conducted using 9,999 permutations and the “ade4” package in R v.3.1.3 (R Core Team 2015). The correlation between geographic and genetic distance was plotted and the correlation coefficient (r) as well as R-squared were estimated using the web version of IBD [29].

### Individual assignment tests and migration

To test the degree of assignment of any individual mosquito to a specific population of origin or an inferred structure-cluster (clusters pre-identified based on the Q matrices retained by CLUMPP [26]), we used the program GeneClass2 v2.0 [30]. The self-assignment tests were performed using the original sampling locality or the clusters identified by STRUCTURE (see results). The same software was used in order to identify the first generation migrants between the major geographic regions of our dataset. To distinguish true from statistical migrants (type I error), we selected the Rannala and Mountain criterion [31]. We used the Monte Carlo resampling algorithm of Paetkau et al. [32], (n = 1,000) to determine the critical value of the L_home/L_max likelihood ratio. Individuals were considered immigrants at two levels of significance, when the probability of being assigned to the reference population was lower than the commonly used 0.05 and lower than 0.01. To complement our results we also used the individual assignment test as implemented in ONCOR software [33].

### Bottleneck effect

Evidence of population bottleneck events was tested using two methods as implemented in the widely used program BOTTLENECK [34]. In the first method, the distribution of the heterozygosity expected from the observed number of alleles is calculated for each population and locus under the assumption of mutation-drift equilibrium. The program provides results under three possible mutation models; the Infinite Allele Model (IAM), the Stepwise Mutation Model (SMM) and the two-phase mutation model (TPM). The SMM is considered better suited for the microsatellite mutation process [35], although in practice, the mutation model that best describes microsatellite evolution varies among loci and falls on a range bordered by the IAM and SMM [34], which are considered the 2 extreme models of mutation [36]. As the two-phase model (TPM) [37] has been considered to better describe microsatellite data here we used both the TPM and the SMM models. Simulation of heterozygosity at mutation-drift equilibrium distributions for the TPM model assumed 70% single-step mutations and 30% of multiple-step mutations, as recommended for microsatellite loci [37]. Significance was assessed using Wilcoxon’s signed rank test, as recommended in the manual for less than 20 markers. The second method we implemented is based on the allele frequency distribution. The program tests whether the allele frequency distribution is approximately L-shaped, which is expected under mutation-drift equilibrium. A shift in the allele frequency distribution is indicative of a recent bottleneck [38]. The bottleneck analysis can only detect extreme reductions in population sizes that have occurred during the last 0.2–4.0 Ne generations [39]. Based on Ne estimations for *Ae*. *aegypti* populations [40–42] and assuming 10 generations per year [2,43,44] the method can detect bottleneck events up to ~50 years ago, but it strongly depends on the study population.

### Inferring population history

**Fig 2:**
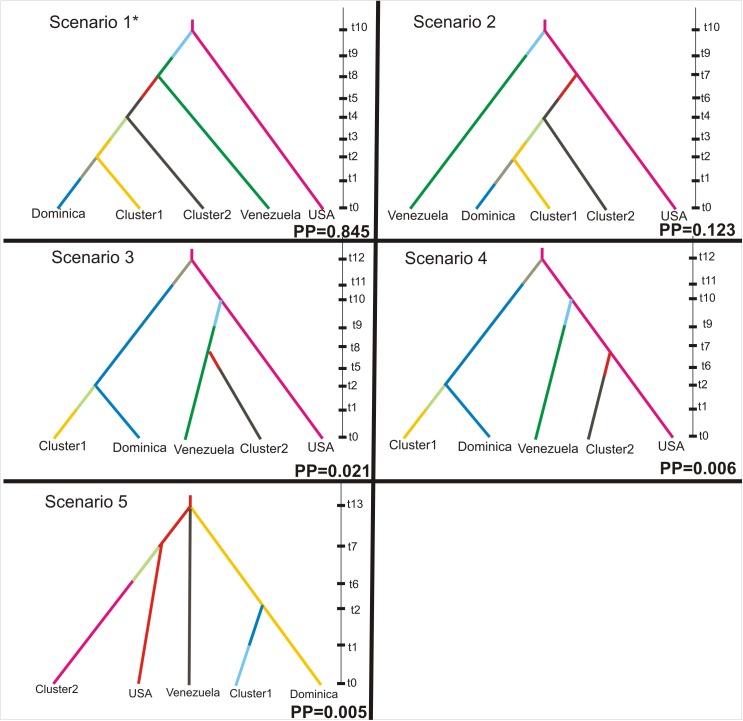
Evolutionary scenarios of *Aedes aegypti* re-invasion in Brazil. Evolutionary scenarios evaluated using Approximate Bayesian Computation (ABC) inference as implemented by the DIYABC software [46]. Scenarios tested included five groups: USA, Venezuela, Dominica, Cluster 2 (northern Brazilian populations), and Cluster 1 (southern Brazilian populations). The time scale on the right of each scenario is the relative time (in generations) with t0 representing the most recent time point and increasing values going back in time. Posterior probabilities (PP) are shown for each scenario. The best supported scenario is indicated by an asterisk (*).

Bayesian computation methods (ABC) [45] as implemented by DIYABC v.2.0.4 [46] were used to infer the population history of *Ae*. *aegypti* in Brazil. For our analysis we used five regions (Venezuela, Dominica, USA, northern, and southern Brazil). Different combinations of these five regions lead to 120 scenarios that could be tested. However, testing all the possible scenarios makes the estimation computationally challenging. Thus, usually [47–49] a small number of scenarios based on historic or other source of data is tested. We chose our scenarios based on (i) the observed population structure, (ii) the results of Monteiro et al. [7], (iii) the historic/epidemiological data (e.g. the first dengue outbreak after eradication recorded in N. Brazil [6]) and (iv) choosing to focus on the origin of the Brazilian populations rather than on the relationships among all five regions. Thus, five possible scenarios were tested (Fig 2). Given that the Dominica population was represented by 48 individuals while the rest of the groups included more than 100 individuals, we randomly subsampled 48 individuals from each one of the remaining groups. Confidence in model choice (how the model fits the observed data) and the best scenario were evaluated as described by the authors [46]. Divergence times were estimated in generations (assuming 10 generations per year; see above) and were assumed to range between 10 and 500 generations (given that after the eradication, *Ae*. *aegypti* was recorded again in Brazil in the late 1970’s). A mutation rate ranging from 9x10^-6^ to 1x10^-5^ was used based on rates reported in the literature for other Diptera species [50,51]. Details on the effective population size and split time between regions used as priors for the ABC analysis are provided in S2 Table.

## Results

### Marker assessment

Sixty Fis values out of 558 (10.7%) population-by-locus comparisons deviated significantly from Hardy-Weinberg equilibrium (HWE) after sequential Bonferroni correction (S3 Table), a level common for microsatellites and most often due to rare null alleles [2,13]. Microchecker indicated that locus A9 has high probability of having null alleles (frequency ranging between 0.08 and 0.3) in 26 out of 48 populations tested. A result that is in agreement with previous findings [13]. Other loci with high probability of null alleles were AC5 and AG1 (with frequency 0.1–0.2), AC4 and AG2 (with frequency 0.06–0.3) in 8, 2, 9, and 7 populations respectively. The remaining loci also exhibited signs of having null alleles but their estimated frequency were lower than 0.16 (S4 Table). A total of 81 out of 2,974 (2.7%) locus-by-locus tests for linkage disequilibrium (LD) (S5 Table) remained significant (p<0.05) after Bonferroni correction for multiple tests, with no loci pair consistently correlated across many populations. This level of LD is low and unlikely to significantly influence methods that assume loci independence.

### Genetic diversity and differentiation

Population genetic statistics for each population are provided in Table 1. Allelic richness (AR) in Brazilian populations was similar to the corresponding values for non-Brazilian populations (Table 1). Private allelic richness (Np) was low (Np< = 0.08) in all cases with the exception of three Brazilian and two non-Brazilian populations (Table 1). Fst values showed moderate levels of population differentiation (considering all 48 populations; mean 0.17; range: 0.01–0.46) with the highest Fst values recorded in Coatzacoalcos (0.21–0.46) and Pance de Cali (0.14–0.46). The genetic differentiation between the Brazilian populations was slightly lower (mean 0.13; range: 0.01–0.29) and it is presented in S6 Table. In all pairwise Fst estimations, Arlequin indicated significant (p<0.05) differentiation.

**Table 1:** Summary of the population genetic diversity statistics for the 48 populations of *Ae*. *aegypti* used in this study.

Population [map code]	Na	mean Na	Na > = 5%	AR(100)	Np(100)	Ho	He	uHe	t test	% loci
Maraba, Brazil [15]	51	4.25	3.00	4.25	0.00	0.52	0.55	0.56	0.10	0.00
Natal, Brazil [18]	47	3.92	2.75	3.92	0.00	0.50	0.50	0.50	0.65	0.00
Aracaju, Brazil [1]	43	3.58	2.92	3.58	0.00	0.45	0.45	0.46	0.53	8.30
Goiania, Brazil [10]	45	3.75	2.83	3.75	0.00	0.46	0.52	0.53	0.12	0.00
Maceio, Brazil [14]	41	3.42	2.33	3.42	0.00	0.41	0.44	0.45	0.29	16.60
Mossoro, Brazil [17]	42	3.50	2.92	3.50	0.00	0.42	0.42	0.43	0.45	0.00
Pau dos Ferros, Brazil [23]	42	3.50	3.00	3.50	0.02	0.43	0.51	0.53	0.07	16.60
Tucurui, Brazil [31]	41	3.42	2.83	3.42	0.00	0.57	0.50	0.52	0.90	16.60
Sao Goncalo, Brazil [28]	47	3.92	3.42	3.92	0.00	0.51	0.53	0.54	0.34	8.30
Cachoeiro, Brazil [4]	45	3.75	2.58	3.75	0.08	0.40	0.40	0.41	0.44	8.30
Cachoeiro, Brazil [5]	49	4.08	3.08	4.08	0.00	0.46	0.49	0.50	0.11	0.00
Jacobina, Brazil [12]	52	4.33	2.75	3.99	0.04	0.43	0.45	0.46	0.16	16.60
Rio de Janeiro, Brazil [25]	49	4.08	2.92	4.08	0.00	0.40	0.45	0.46	0.15	16.60
SJR Preto, Brazil [29]	48	4.00	3.17	4.00	0.00	0.42	0.50	0.50	0.03	33.30
Santos, Brazil [27]	59	4.92	3.58	4.92	0.08	0.45	0.50	0.51	0.13	8.30
Rio Branco, Brazil [24]	49	4.08	3.17	4.08	0.00	0.48	0.50	0.51	0.24	16.60
Parnaiba, Brazil [21]	50	4.17	2.42	4.17	0.25	0.33	0.44	0.45	0.00	25.00
Pacaraima, Brazil [20]	44	3.67	2.75	3.67	0.00	0.46	0.50	0.51	0.17	8.30
Montes Claros, Brazil [16]	42	3.50	3.17	3.50	0.00	0.51	0.52	0.53	0.34	8.30
Itacoatiara, Brazil [11]	39	3.25	2.83	3.25	0.00	0.42	0.49	0.50	0.10	16.60
Foz do lguacu, Brazil [9]	58	4.83	3.67	4.83	0.25	0.62	0.58	0.59	0.96	0.00
Fortaleza, Brazil [8]	42	3.50	2.83	3.50	0.00	0.41	0.44	0.45	0.27	16.60
Castanhal, Brazil [7]	47	3.92	3.58	3.92	0.00	0.48	0.53	0.54	0.13	25.00
Boa Vista, Brazil [3]	41	3.42	2.25	3.42	0.00	0.38	0.42	0.42	0.09	8.30
Belem, Brazil [2]	50	4.17	3.25	4.17	0.17	0.48	0.55	0.56	0.03	25.00
Tocantins, Brazil [30]	50	4.17	3.08	4.17	0.00	0.47	0.50	0.51	0.20	8.30
Parnamirim, Brazil [22]	57	4.75	2.83	4.66	0.08	0.47	0.53	0.54	0.04	16.60
Macapa, Brazil [13]	50	4.17	3.08	4.09	0.08	0.51	0.55	0.55	0.07	8.30
Campo Grande, Brazil [6]	47	3.92	2.42	3.89	0.00	0.39	0.44	0.44	0.01	0.00
Nova Iguacu, Brazil [19]	54	4.50	2.58	4.34	0.00	0.39	0.46	0.47	0.05	8.30
Santarem, Brazil [26]	50	4.17	3.00	3.93	0.00	0.53	0.54	0.55	0.22	0.00
Puerto Rico, Caribbean [46]	57	4.75	3.83	4.74	0.08	0.60	0.59	0.60	0.60	0.00
Pance de Cali, Colombia [32]	49	4.08	3.50	4.08	0.17	0.56	0.58	0.59	0.30	0.00
Cali, Colombia [33]	33	2.75	2.42	2.72	0.00	0.40	0.42	0.42	0.30	33.30
Tijuana, Mexico [42]	35	2.92	2.92	2.92	0.00	0.63	0.55	0.56	0.98	8.30
Key West, USA [37]	61	5.08	3.75	5.06	0.00	0.61	0.61	0.61	0.55	8.30
Amacuzac, Mexico [39]	48	4.00	3.00	3.99	0.08	0.57	0.53	0.53	0.98	16.60
Costa Rica [43]	55	4.58	3.33	4.58	0.00	0.55	0.61	0.61	0.03	8.30
Trinidad, Caribbean [48]	53	4.42	3.58	4.41	0.08	0.47	0.53	0.54	0.01	41.60
Puerto Rico, Caribbean [45]	53	4.42	3.67	4.40	0.00	0.57	0.54	0.54	0.15	41.60
Carriacou, Caribbean [47]	31	2.58	2.50	2.58	0.17	0.72	0.51	0.52	0.00	41.60
Dominica, Caribbean [44]	41	3.42	3.08	3.42	0.08	0.44	0.44	0.45	0.45	16.60
Pijijapan, Mexico [41]	40	3.33	2.92	3.33	0.00	0.39	0.46	0.46	0.06	16.60
Coatzacoalcos, Mexico [40]	31	2.58	2.50	2.58	0.00	0.42	0.34	0.34	0.02	16.60
Bolivar, Venezuela [34]	52	4.33	3.33	4.33	0.00	0.52	0.55	0.55	0.42	8.30
Zulia, Venezuela [35]	49	4.08	3.33	4.08	0.00	0.55	0.53	0.54	0.20	8.30
Houston, USA [36]	40	3.33	2.58	3.33	0.00	0.55	0.45	0.46	0.02	16.60
Miami, USA [38]	65	5.42	3.83	5.42	0.08	0.67	0.63	0.64	0.16	0.00

Abbreviations: Na: total number of different alleles, Na > = 5%: mean number of different alleles with a frequency > = 5%, AR(100): allele richness estimated by rarefraction (N = 100 genes), Np(100): number of private alleles estimated by rarefraction (N = 100 genes), Ho: observed Heterozygosity, He: expected Heterozygosity, uHe: Unbiased Expected Heterozygosity, t test: p values resulted from paired t test comparing the Ho and He across all loci (p<0.05 considered significant), %loci: percentage of loci out of HWE, as determined by the Fis significant values after Bonferroni correction.

### Genetic structure

We first performed a preliminary STRUCTURE analysis (Fig 1B) on the complete data set of all 48 populations. This analysis confirmed the presence of the two genetic clusters (Fig 1) and the approximate border between them (Fig 1A) which runs from east central Brazil (northern to Parnaiba (21)) through the region between Tocantins (30) and Maraba (15) and ends in North-West Brazil. The southern cluster included 23 Brazilian and one Dominican populations (here called “Cluster 1”). The northern cluster (“Cluster 2”) included 25 populations from the Caribbean, North/Central America, and the northern parts of South America, including North Brazilian populations. Three populations (Itacoatiara, Tocantins and Santos) located in central Brazil (forming a path from north-central to south-central) appeared admixed ancestry (Fig 1) since their Q values to their cluster were low (0.56–0.62) and many of the individuals were not assigned to the respective cluster (Santos; 33% of the samples had Q values <0.40 for Cluster 1, Itacoatiara; 40% of the samples had Q values <0.40 for Cluster 2, Tocantins; >40% of the samples had Q values <0.23 for Cluster 1).

**Fig 3:**
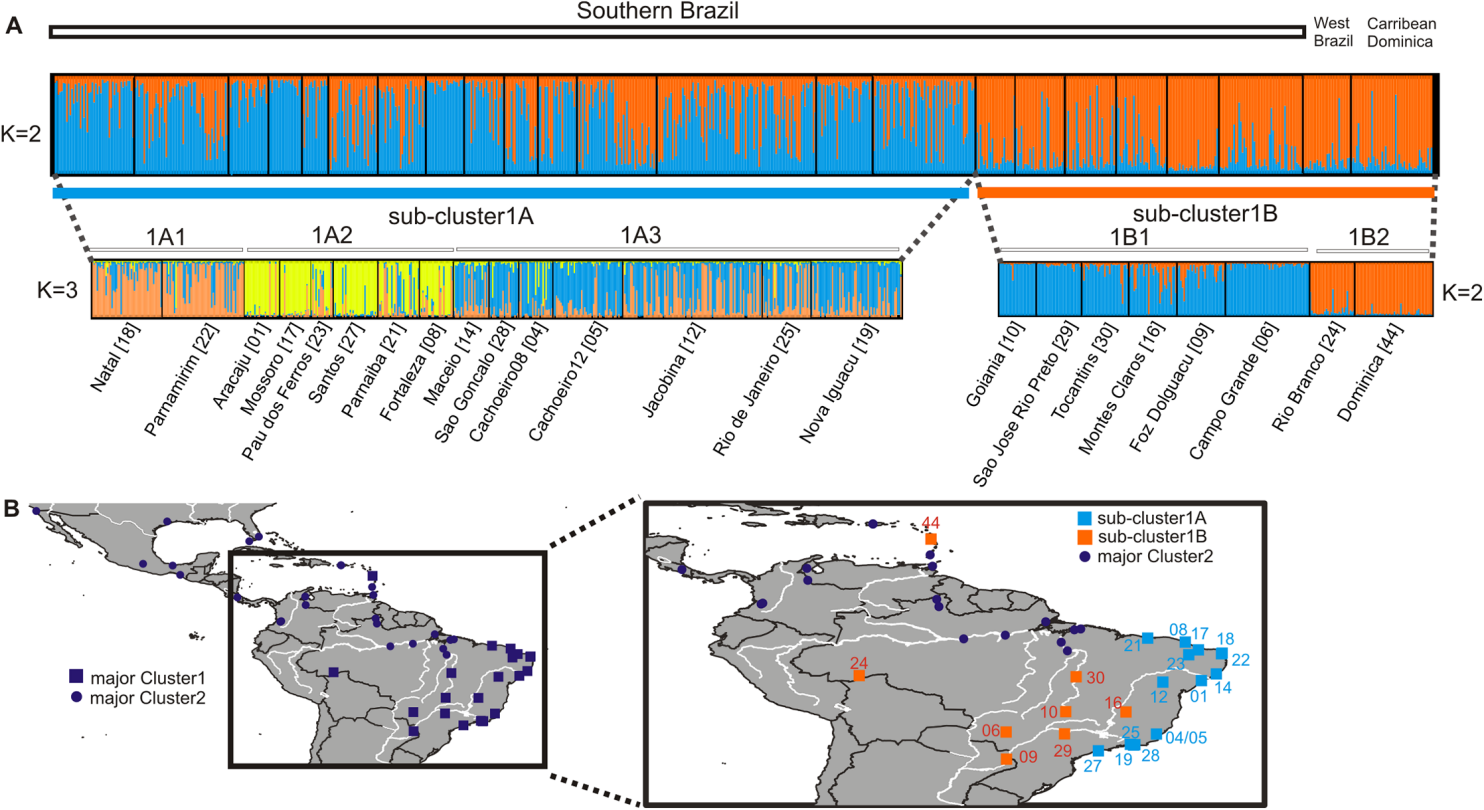
Genetic structure analysis for Cluster 1. STRUCTURE bar plots for *Ae*. *aegypti* populations of southern Brazil-Dominica data set (A). See legend of Fig 1B for details. The genetic clusters as indicated by the Evanno method are plotted. Populations’ code numbers in brackets are referred to the map codes in B and as in S1 Table. The distribution of the Clusters populations (Cluster 1; squares and Cluster 2; dots) as indicated by the preliminary STRUCTURE analysis with focus on the sub-clusters (sub-clusters 1A; light blue and 1B; orange) found within Cluster 1 populations is presented (B). The main rivers in the area are presented.

We then carried out analyses on each of the two clusters separately. Fig 3A shows the result of the Structure analysis on Cluster 1. The Evanno et al. [24] method identified K = 2, as the most likely number of sub-clusters (sub-clusters 1A and 1B). The two genetic sub-clusters seemed to be well differentiated with little overlap between their DAPC defined clouds (S1 Fig). Sub-cluster 1A includes eastern and south-central populations and sub-cluster 1B includes the western Brazil populations and Dominica (Fig 3B). A STRUCTURE analysis on the two sub-clusters separately revealed further genetic structure (Fig 3A), with sub-cluster 1A consisting of three smaller genetic groups (1A1, 1A2, 1A3), and sub-cluster 1B consisting of two groups (1B1 and 1B2).

**Fig 4:**
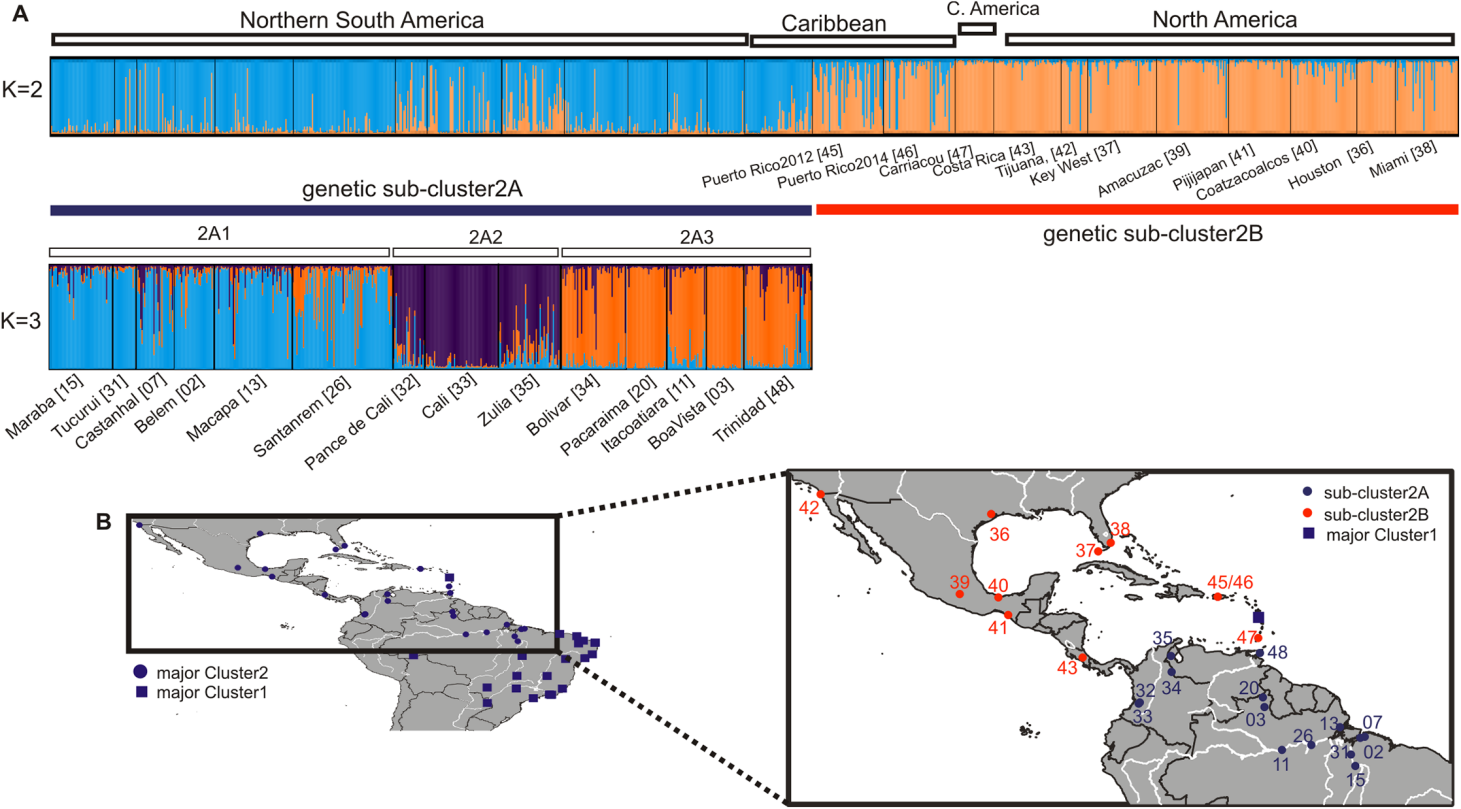
Genetic structure analysis for Cluster 2. STRUCTURE bar plots for the *Ae*. *aegypti* populations of Cluster 2 data set (A). See legend of Fig 1B and Fig 3 for details. The distribution of the major Clusters with focus on the two sub-clusters (sub-clusters 2A; light blue and 2B; orange) found within Cluster 2 populations is presented (B).

For Cluster 2 the Evanno et al. [24] method also identified K = 2 as the most likely number of clusters (Fig 4A): sub-cluster 2A to the South and sub-cluster 2B to the North (Fig 4B). These two groups were well differentiated and only slightly overlapped (S1 Fig) in their DAPC clouds. Given that the genetic substructure of North/Central America-Caribbean cluster has been discussed elsewhere [2], we focused our further analyses on sub-cluster 2A for which the Evanno et al. [24] supported the presence of three sub-groups (2A1, 2A2 and 2A3).

The assignment of the samples from each population to each one of the three DAPC-groups (S2 Fig) is presented in detail in S7 Table. Considering Cluster 1, the majority of the samples from sub-clusters 1A2 and 1A3 (south-eastern) were assigned to DAPC group1, from sub-cluster 1B1 (south-western) to group2 and from 1B2 (western) to group3 (S7 Table). The DAPC-groups for Cluster 2 (S2 Fig) confirmed the separation of North/Central America-Caribbean (DAPC-group2) from South American populations (DAPC-groups 1 and 3) (S2 Fig and S7 Table) with the exception of Coatzacoalcos (Mexico) population, which is included in DAPC-group1 instead of DAPC-group2 (S7 Table).

Mantel test on Cluster 1 dataset (S3 Fig) supported the presence of Isolation by Distance (IBD) between all pairs of populations in Cluster 1 (r = 0.55; p = 0.001) as well as for the populations within the sub-cluster 1B (r = 0.69; p = 0.008) but not within 1A (r = 0.12; p = 0.064). For Cluster 2, although the Mantel test supported (p<0.05) the presence of Isolation by Distance (IBD) for both Cluster 2 and the sub-cluster 2A (S3I Fig), the linear relationship between the genetic and the geographic distance was weak (r = 0.24 and r = 0.28 respectively).

Levels of genetic differentiation (Fst values) between all pairs of populations within Cluster 1 and Cluster 2 are presented in S6 Table and between broader regions (Caribbean, USA, Colombia, Venezuela, Mexico, Costa Rica and Brazilian Clusters 1 and 2) in S8 Table. Brazilian Cluster 1 and Cluster 2 populations showed low to moderate levels of genetic differentiation with Fst values, within each cluster, up to 0.21 and 0.22 for Cluster 1 and Cluster 2, respectively.

Pooling the samples within each genetic cluster or group of samples within a country (Caribbean, USA, Colombia, Venezuela, Mexico, Costa Rica and Brazilian Clusters 1 and 2), revealed high private allele (Np) richness, especially in Cluster 1 (S8 Table).

When we consider STRUCTURE Clusters 1 and 2 and the populations from the non-eradicated areas (Caribbean, Venezuela and southern part of North America) as three separate groups, ANOVA results (S9 Table) showed that the eradicated areas (Clusters 1 and 2 of Brazil) have significantly lower mean Ho levels (ANOVA; F_2,38_ = 12.5; p<0.001). Nevertheless, no significant difference was found in allelic richness levels (S9 Table).

S10 Table shows the results of the AMOVA analyses. Most of the genetic variation occurred within populations (81% and 71% for populations within Clusters 1 and 2, respectively), while variation among populations was less than 20% for Cluster 1 and less than 30% for Cluster 2. Considering the structure-defined sub-clusters instead of the original populations, the percentage of among groups variation decreased even more, depending on the number of genetic clusters considered (S10 Table).

### Assignment tests and indications of mixed ancestry

S7 Table shows the results of the GeneClass2 assignments. Only 47.8% of the individuals in Cluster 1 were correctly assigned back to their population of origin when using the original populations as a reference. This percentage increased when the STRUCTURE inferred clusters, rather than the populations, were used as reference (76.9% for K = 2; 62.3% for K = 5). A larger percentage of individuals in Cluster 2 were correctly assigned back to their population of origin (55.4%) or to their cluster (88.6% for K = 2; 86.1% for K = 4).

### Detection of first generation migrants

S11 Table shows the number of individuals within each group of populations that could be first generation migrants according to the Geneclass2 results, at two levels of significance (p<0.01 and <0.05). Focusing on the two Brazilian clusters, the majority of the individuals that could be first generation migrants within Cluster 1 originated from Cluster 2. Moreover, most of them were from regions close to the border between the two Brazilian Clusters (Tocantins, Parnaiba) and Santos, which belongs to the admixed zone (Fig 1B). The majority of the individuals that could be first generation migrants within Cluster 2, and originated from Venezuela (S11 Table), were found in a single Brazilian population (Pacaraima; map code 20; Fig 1A and Fig 4B). The ONCOR results presented on the lower part of S11 Table are in agreement with the Geneclass2 results.

### Testing for bottleneck effect

S12 Table shows the results of the bottleneck analyses. Using the TPM model the BOTTLENECK results were significant (p<0.05) for 21 populations (8 from Brazil). Using the SMM model only two non-Brazilian populations showed signs of significant demographic changes. Mode shift in allele frequency distributions was present in four non Brazilian populations (S12 Table).

### Inferring Brazilian population history

Approximate Bayesian Computation (ABC) analysis provided strong support (PP = 0.845) for scenario 1 (Fig 2). In this scenario, *Ae*. *aegypti* from the non-eradicated populations from Venezuela invaded North Brazil (Cluster 2) and subsequently invaded the South of Brazil (Cluster 1). Except for scenario 2 (PP = 0.12), alternative scenarios tested were poorly supported (Fig 2; PP<0.022). In particular, the scenario favored by Monteiro et al. [7] (Scenario 3) has little support testifying to the importance of the additional information provided by the larger numbers of samples analyzed in this paper. S2 Table shows the posterior distribution of parameters for Approximate Bayesian Computation (ABC) analysis using the DIYABC software [46]. The estimated mutation rate under the best-fit scenario was 9.5x10^-6^, and falls within the range of microsatellite mutation rates estimated for other Diptera [50,51].

## Discussion

### Genetic structure and gene flow between the major clusters

Our results show clear genetic differentiation between two groups of Brazilian *Ae*. *aegypti* populations (southern-Cluster 1 and northern-Cluster 2), that are roughly separated (except Rio Branco; map code 24; Fig 1A) by the Amazon forest. Our analyses confirmed previous findings [7,8,10,52,53] and revealed also an admixture zone between those clusters (Fig 1 and S7 Table). The admixed ancestry is expected for Santos, given that it is the principal port in Brazil as well as on a major trucking route.

Our results also provide insights into the patterns and levels of genetic connectivity between and within these two population clusters, revealing gene flow across the landscape. The detection of possible first generation migrants, the large number of Cluster 1 individuals that are assigned to Cluster 2 (S11 Table), and the moderate levels of genetic differentiation of these two clusters (S6 and S8 Tables) support the existence of genetic connectivity despite genetic distinctiveness (Fig 1B). This apparent contradiction can be resolved by noting that the majority of gene flow occurs in the areas close to the geographic borders of these two clusters rather than between distant localities. For instance, most of the possible first generation migrants detected in Cluster 1 coming from Cluster 2, are from sites in the admixture zone or close to the border between the two Clusters. Results from the Mantel test support the existence of a correlation between genetic and geographic distances (S3 Fig), which implies an ongoing gene exchange between geographically close samples. This gene exchange is less intense at larger geographic scales, where the existence of distinct genetic clusters implies the presence of limitations to gene flow.

### The Southern Brazil-Dominica group

Focusing on Cluster 1, two major genetic groups exist; the southeastern and the southwestern (Fig 3), a separation also supported by RAPD markers [8]. The strong geographic pattern is supported by the Isolation By Distance results (S3 Fig), a finding also reported by other studies [8,54] and by the DAPC analysis (S7 Table).

While genetically distinct, many populations, especially those in sub-cluster 1A, show signs of mixed ancestry (S7 Table). This is further corroborated by results of the DAPC analysis (S2 Fig) and the assignment tests, where a low percentage of individuals is assigned back to their population of origin (S7 Table). For example, Jacobina individuals have an admixed genetic profile, as suggested by their Q values (S7 Table), by the fact that they are found in all three DAPC-defined groups, and by their low percentage of correct assignment to their population of origin that is < 30% (S7 Table). Additionally, the private allele richness (Np) was low in almost all populations of Cluster 1, supporting the evidence of either admixed ancestry or gene flow among populations.

Within Cluster 1 we also found patterns of genetic connectivity that are not correlated with geographic distances, suggesting that the main driver of *Ae*. *aegypti* dispersal in these cases is human transport. This is especially true for sampling sites in the Rio Branco (see clustering in Fig 3), Foz do lguaçu, and Rio de Janeiro areas (see Fig 3 and Fst values in S6 Table). Foz do lguaçu samples have also a high proportion of private alleles (Np = 0.25, S6 Table), indicating limited gene flow. The high value of Np in Foz do lguaçu is possibly explained by the fact that it is located at the border between Brazil and Paraguay and Argentina (Fig 1A). The Rio de Janeiro samples (Fig 3) exhibit high levels of genetic variability, admixed ancestry, and no clear assignment to a DAPC group (S8 Table). One possible explanation is that this region is a cross-road, where mosquitoes from different places in Brazil and from abroad converge and then disperse again, due to the extensive road network that connects this area to all other large cities in Brazil and the heavy goods and tourist traffic of the area. Interestingly, this reasoning also explains the fact that Rio de Janeiro and its neighboring areas are the most frequent entry points for dengue into Brazil and that DENV-2 infection rates are very heterogeneous [54].

### Northern South America cluster

The second major genetic cluster in Brazil (sub-cluster 2A) is genetically close to Colombia, Venezuela, and Trinidad samples (Fig 4). The correlation between genetic and geographic distances is not as strong for this cluster as in Cluster 1 (S3 Fig). The low proportion of private alleles (Np; S6 Table) in Cluster 2 populations suggests that there is gene flow among them. Goncalves da Silva et al. [55] using mtDNA data also detected extensive gene flow among the major cities in this area. The occurrence of gene flow but with limited IBD may be explained by the different topography of the region. Most of the sampling sites in this study are connected by rivers including the Amazon (Fig 4B). Boat traffic along this water body may facilitate *Ae*. *aegypti* dispersal [56,57] in ways that will obscure patterns of correlation between genetic and geographic distances.

Our data show high levels of gene flow between Venezuela and Brazilian Cluster 2 samples. This is supported by the high number of possible first generation migrants detected in Brazilian Cluster 2 (S11 Table) and the moderate values of Fst (S6 Table) between these two areas. However, gene flow seems to be primarily among geographically close populations, since most of the possible first generation migrants detected in Brazilian Cluster 2 are found in Pacaraima and Boa Vista (Fig 1A), on the Brazilian border with Venezuela. There is intense traffic between Venezuela and these two cities. Also the genetic assignment of Boa Vista (with Bolivar (Venezuela); Fig 4) is particularly interesting because Boa Visa is considered an important entry point for new serotypes and genotypes of dengue virus into Brazil from the northern South American countries [54]. The genetic connection we found between North Brazil and Venezuela is consistent with other studies [54,55] and further support the hypothesis that *Ae*. *aegy*pti populations from northern Brazil derive from Venezuela, where this species was never eradicated [7]. Boa Vista was the site of the first dengue (DENV-1 and DENV-4) outbreak after eradication (1981–1982), which did not spread to the rest of the country for several years; DENV-1 was first reported in Rio de Janeiro in 1986 [58]. In 2000, Boa Vista also reported DENV-3 about the same time DENV-3 was reported in Rio de Janeiro. Given that all dengue serotypes have been reported Venezuela, it is more likely that DENV-3 was introduced in Boa Vista from Venezuela and then spread south to Rio de Janeiro, rather than the other way around.

### Inferring the population history of *Ae*. *aegypti* in Brazil

What were the origins of the two major genetic clusters in Brazil? Brazil, North America, and the Caribbean, have a long history of international trade and populations of *Ae*. *aegypti* with close genetic affinities [1,7,59]. Here the inclusion of three Caribbean populations reveals further complexity in the genetic affinities of *Ae*. *aegypti* in the Americas in agreement with previous findings [2]. For instance, samples from Dominica, Trinidad and Carriacou, although geographically close, are genetically distinct as they belong to three different groups (Trinidad groups with Venezuela-Colombia-northern Brazil, Dominica groups with southern Brazil and Carriacou groups with North/Central America; Fig 1 and Fig 4B).

To shed light on demographic dynamics, origin(s), and timing of *Ae*. *aegypti* re-infestation in the 1970s [6,7], we tested for the relative likelihoods of alternative colonization scenarios using Approximate Bayesian Computation, ABC (Fig 2). If we assume that *Ae*. *aegypti* survived in residual small populations, a genetic signature should be detectable, such as lower genetic diversity than samples from non-eradicated areas and signs of recent bottlenecks. Our data do not support this hypothesis; Brazilian samples have levels of genetic diversity comparable with that found in samples from countries where eradication never occurred (Table 1 and S9 Table). As far as the signs of bottleneck are concerned, although our analyses indicated 8 (one of them marginally significant) out of 31 Brazilian populations exhibiting signs of bottleneck (S12 Table), this was supported by only one method and one model. Stronger signs of bottleneck would be expected if small populations had survived the eradication in refugia, although the invasion hypothesis also assumes some level of bottleneck. Combining all the analyses we are in favor of the hypothesis supporting a re-invasion from the neighboring non-eradicated areas, although we cannot completely rule out the hypothesis of refugia or a combination of both.

The ABC analyses (Fig 2 and S2 Table), strongly supported Venezuela as the origin of the northern Brazilian samples, and followed by a southern expansion into Brazil. The divergence times estimates provided by the ABC analyses (S2 Table) are consistent with *Ae*. *aegypti* re-infesting northern Brazil in the 1970s [6] and spreading to the South during the late 1980s [6], as suggested by the timing the dengue outbreaks that occurred at those times. This scenario is also supported by the gene flow data between Venezuela and North Brazil and between North and South Brazil detected here (Fig 1, Fig 4 and S11 Table) and in other studies [7,55]. However, the ABC analysis did not support the suggestion from Monteiro et al. [7] that the southern Brazil Cluster originated from Dominica, instead suggesting northern Brazil as the most likely source (Fig 2) for the Southern Brazil cluster.

### Conclusions and summary

Consistent with a number of previous studies, there are two major genetic groups of *Ae*. *aegypti* populations in present day Brazil, a northeast set and south set (Fig 1). The most likely scenario of establishment of these groups after eradication ~60 years ago is recolonization from Venezuela where eradication was never achieved. This recolonization occurred in two waves, first in the north of Brazil then later in the south. The genetic differentiation of the two groups likely occurred when the first establishment in the south from the north occurred in a coastal city and then gradually expanded northward and inland. The present boundary between these groups may be due to environmental factors or simply an historical pattern that may disappear with time; hybridization in the border area indicates no barriers to gene flow. We confirm that the Caribbean island Dominica, is genetically closely related to the south Brazil genetic group, but contrary to a previous study, our more detailed analysis indicates that Dominica was likely established from Brazil.

Within each of the two major genetic groupings, further genetic subdivision is detectable. The level of gene flow among these subdivisions and individual populations is complex and varies by region. In some cases isolation by distance can be detected while in other areas not, likely due to whether migration is “natural” or human induced. Given the large area and heterogeneity of Brazil, this heterogeneity in genetic patterns and processes is to be expected.

While our present work establishes a reasonably good overview of history and patterns of genetic diversity of *Ae*. *aegypti* in Brazil, it is only the first step in fuller understanding of the population biology of this major vector in a country plagued for centuries by diseases it transmits. While fundamental, genetic data alone provide limited inferences of processes at work incorporating detailed information on landscape features such as highways, rivers, shipping routes, etc., overlaid with genetic data should provide more insights into processes. It is the understanding of contemporary processes, especially levels and patterns of movement among populations that are most useful in designing control measures.

## Supporting information

S1 Fig Discriminant Analysis of Principal Components (DAPC) on the STRUCTURE-predefined clusters. DAPC on the two genetic sub-clusters as predefined according to the STRUCTURE output for K = 2 for Cluster 1 (**A**) and Cluster 2 (**B**) populations.
(TIF)

S2 Fig Discriminant Analysis of Principal Components (DAPC) for Clusters 1 and 2. DAPC scatterplots representing three groups of populations for each Cluster 1 (**A**) and Cluster 2 (**B**). The graphs represent the individuals as dots and the groups as inertia ellipses. DA eigenvalues of the analysis are displayed in insets.
(TIF)

S3 Fig Isolation-by-distance. Isolation-by-distance plots for all pairs of populations within the STRUCTURE defined Clusters 1 (**A, B**) and 2 (**G, H**) and all populations pairs within the sub-cluster 1A (**C, D**), sub-cluster 1B (**E, F**) and sub-cluster 2A (**I, J**). Statistical significance was evaluated through Mantel test as implemented using the ade4 package in R. The original value of the correlation between the two matrices (geographic distance and genetic distance-Fst values) is represented by a dot, while the histograms represent the permutated values assuming absence of spatial structure. Significant spatial structure result in the original value being out of the reference distribution. The correlation between geographic and genetic distance was plotted and the correlation coefficient (r) and as well as the R-squared, were estimated using the web version of IBD.
(TIF)

S1 Table Population information for the *Ae*. *aegypti* samples used in this study. Numbers in brackets after the population name are as in Fig 1. Abbreviations: Code: population code for the downstream analyses, Year: year of collection, Gen lab: number of generations in laboratory conditions, N: number of samples.
(DOCX)

S2 Table Parameters of Approximate Bayesian Computation (ABC) analysis. Prior and posterior distribution of parameters of Approximate Bayesian Computation (ABC) analysis, to test hypothesis of re-colonization of Brazil by *Ae*. *aegypti*, using DIYABC software [46]. Priors for splitting time are all uniform distributions and those for population size are log-uniform. Posterior probabilities for each scenario depicted in Fig 2 are shown with 95% confidence intervals. Error and posterior distributions of parameters (median with 90% confidence interval) are reported for the best-fit scenario (Scenario 1 in Fig 2). Summary statistics used to compare simulated and observed data sets were: mean number of alleles, mean genetic diversity [60], mean size variance, and Fst. Divergence times are measured in generations.
(DOCX)

S3 Table *Aedes aegypti* Fis values per locus and per population. Weir & Cockerham's [61] F_IS_ estimated values using the Genepop software [15] for all *Ae*. *aegypti* populations for each microsatellite locus. Values that remained significant (alpha = 0.05) after sequential Bonferroni correction are indicated by bold characters. Abbreviations: NA: non available.
(DOCX)

S4 Table Null allele frequencies. Null allele frequencies for each of the 12 microsatellite markers and the populations with at least one possible null allele at a given locus as estimated using the Microchecker software [20].
(DOCX)

S5 Table Linkage Disequilibrium (LD) test. Locus-by-locus test for Linkage Disequilibrium (LD) for all 48 populations under study as implemented in Genepop software [15]. Only cases that remained significant after Bonferroni correction (p<0.05) for multiple tests are presented. Numbers in brackets after the population name are as in S1 Table and Fig 1A.
(DOCX)

S6 Table Pairwise Fst values. Pairwise Fst values between all Brazilian *Ae*. *aegypti* populations as estimated by FreeNA [22]. Fst values without correction are shown below diagonal while the FreeNA corrected values are shown above diagonal. All pairwise comparisons were significant (p<0.05) in Arlequin estimates. Population codes are as in S1 Table.
(XLSX)

S7 Table Summary of the Q values for STRUCTURE-defined groups, assignment to DAPC-groups and result from assignment tests for all populations and groups of the study. Assignment of all *Ae*. *aegypti* samples to the genetic groups based on the Q-matrix retrieved from CLUMPP [26] for each STRUCTURE-defined group (in light gray Major Cluster1; Clusters 1A, 1B; sub-clusters 1A1, 1A2, 1A3, 1B1, 1B2 and in dark gray Major Cluster2; Clusters 2A, 2B; sub-clusters 2A1, 2A2, 2A3) and each DAPC-inferred group (Group1, Group2, Group3). Cases exhibiting admixed ancestry (0.50<Q value<0.62) based on the STRUCTURE analyses, are indicated by bold characters and Q values for all the structure groups are provided. In the last column the percentage (%) of correct assignment (alpha = 0.01) back to the reference population or defined STRUCTURE-cluster as estimated by the Geneclass2 [30] is presented. Population codes are as in S1 Table and Fig 1. Abbreviations: Pop. Q value: Q values retained for each population from CLUMPP which summarizes the Q values form the 10 independent STRUCTURE runs, Ind. Range: Range for the Q values retained for each individual mosquito, Pop: population, N: number of individuals.
(PDF)

S8 Table Fst values and private allele richness between *Ae*. *aegypti* populations pooled in geographic groups or in STRUCTURE defined clusters. Fst values as estimated by FreeNA (below diagonal) and Number of private allele richness (Np) (on diagonal in bold type) as estimated by HPrare (assuming 100 genes) between *Ae*. *aegypti* populations pooled in geographic groups (1–8) or in STRUCTURE defined clusters (9–10). All Fst values were significant in Arlequin estimates (p<0.05).
(DOCX)

S9 Table ANOVA analyses of populations within Cluster 1 or Cluster 2 and the non-eradicated areas. F scores and p values of the One-way ANOVA applied for the Heterozygosity, Private alleles, number of alleles and Allelic richness. In cases of statistical significance (p<0.05) post-hoc Tukey and Bonferroni tests were applied. Statistical significant cases (p<0.05) are indicated by bold characters.
(DOCX)

S10 Table Within groups variation (AMOVA). The percentage of within groups variation as estimated by AMOVA in the GenAlex software for different levels of grouping for the two Structure-defined Clusters 1 and 2 as in Fig 3 and Fig 4, respectively.
(DOCX)

S11 Table First generation migrants estimated by Geneclass2 and assignment test results estimated by ONCOR. First generation migrants (Nmigrants) for *Ae*. *aegypti* populations between the major geographic regions of the study area and genetically defined clusters, as estimated by Geneclass2 [30] considering two levels of significance (p<0.05 and <0.01). The most possible source of migrants is also presented. At the lower part of the Table the assignment of individuals to groups according to ONCOR is presented. Individuals assigned to “their own” group are underlined.
(DOCX)

S12 Table Evidence for bottleneck. Results of the bottleneck tests for populations for which at least one method indicated significance population reductions. Results from both Wilcoxon test (TPM and SMM model) and the mode shift in allele frequency distributions are presented, as estimated by BOTTLENECK [34]. Significant values (p<0.05) are indicated with bold characters.
(DOCX)

S1 Appendix Raw data used in the study. (XLSX)
