# Supplementary figures and images for: Tracking the return of Aedes aegypti to Brazil, the major vector of the dengue, chikungunya and Zika viruses
Source: PLoS Negl Trop Dis. 2017 Jul 25;11(7):e0005653. doi: 10.1371/journal.pntd.0005653 (PMC5526527; doi:10.1371/journal.pntd.0005653)

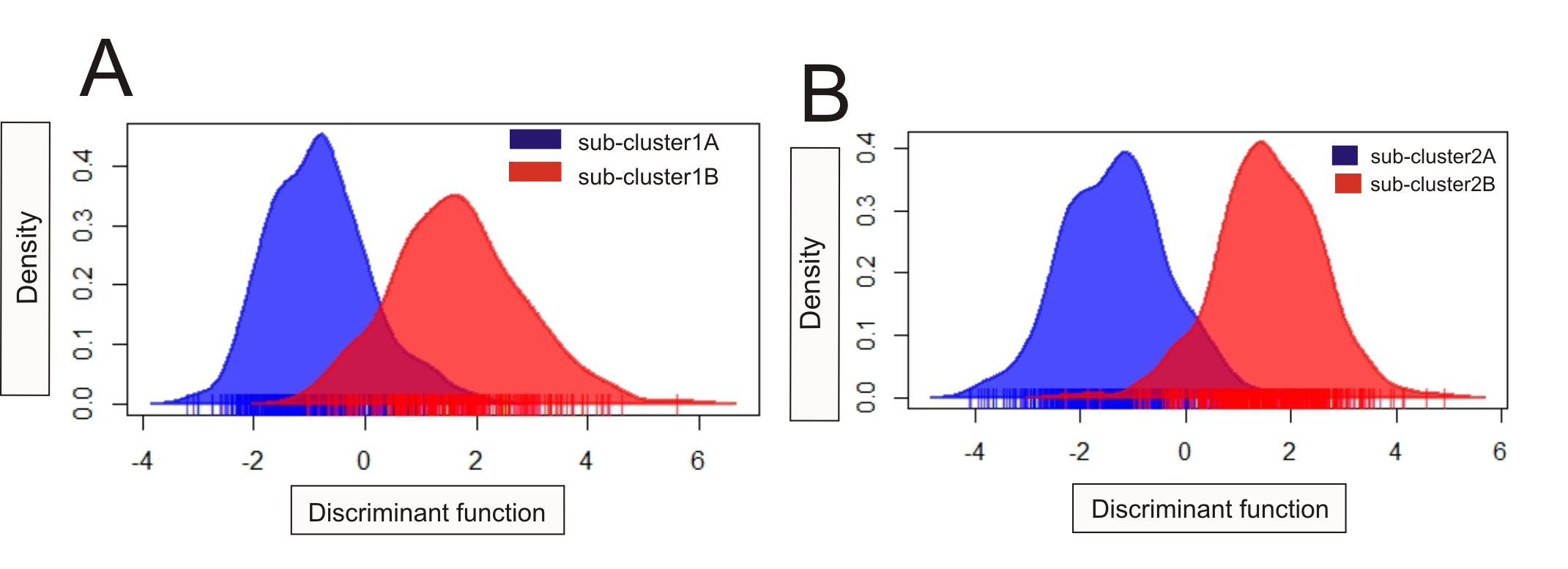

Supplement: S1 Fig — DAPC on the two genetic sub-clusters as predefined according to the STRUCTURE output for K = 2 for Cluster 1 (A) and Cluster 2 (B) populations. (TIF) [file pntd.0005653.s001.tif]

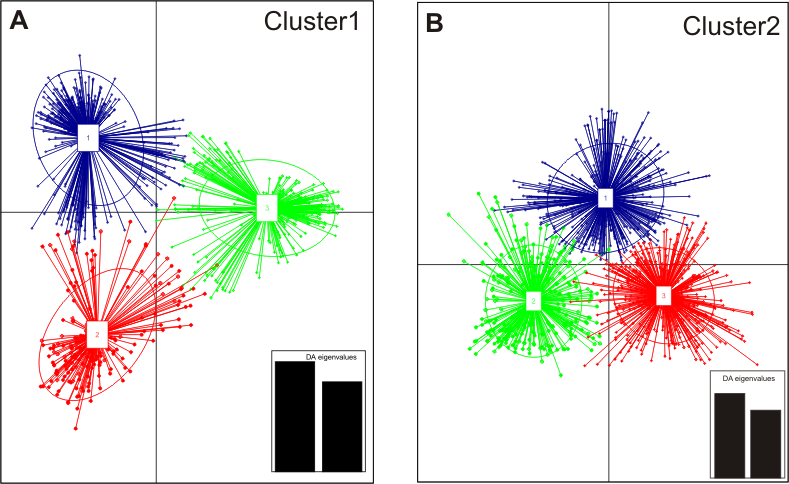

Supplement: S2 Fig — DAPC scatterplots representing three groups of populations for each Cluster 1 (A) and Cluster 2 (B). The graphs represent the individuals as dots and the groups as inertia ellipses. DA eigenvalues of the analysis are displayed in insets. (TIF) [file pntd.0005653.s002.tif]

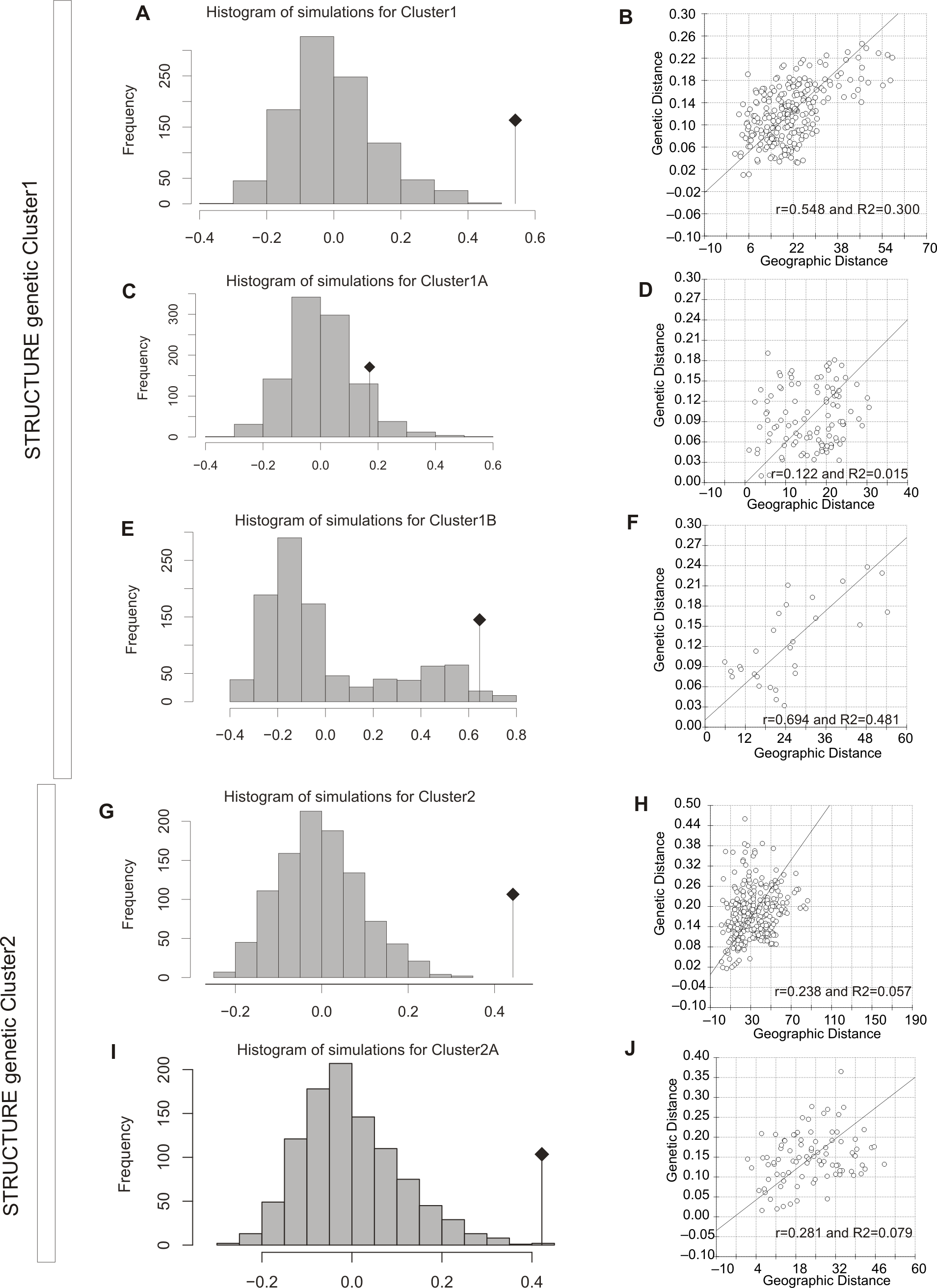

Supplement: S3 Fig — Isolation-by-distance plots for all pairs of populations within the STRUCTURE defined Clusters 1 (A, B) and 2 (G, H) and all populations pairs within the sub-cluster 1A (C, D), sub-cluster 1B (E, F) and sub-cluster 2A (I, J). Statistical significance was evaluated through Mantel test as implemented using the ade4 package in R. The original value of the correlation between the two matrices (geographic distance and genetic distance-Fst values) is represented by a dot, while the histograms represent the permutated values assuming absence of spatial structure. Significant spatial structure result in the original value being out of the reference distribution. The correlation between geographic and genetic distance was plotted and the correlation coefficient (r) and as well as the R-squared, were estimated using the web version of IBD. (TIF) [file pntd.0005653.s003.tif]
