# Supplementary material for: Tracking the return of Aedes aegypti to Brazil, the major vector of the dengue, chikungunya and Zika viruses
Source: PLoS Negl Trop Dis. 2017 Jul 25;11(7):e0005653. doi: 10.1371/journal.pntd.0005653 (PMC5526527; doi:10.1371/journal.pntd.0005653)
Supplement: S1 Table — Numbers in brackets after the population name are as in Fig 1. Abbreviations: Code: population code for the downstream analyses, Year: year of collection, Gen lab: number of generations in laboratory conditions, N: number of samples. (DOCX) [file pntd.0005653.s004.docx]

**Table S1. Population information for the *Ae. aegypti* samples used in this study.**

| **Code** | **Locality [map code]** | **Country** | **Region** | | **Latitude** | **Longitude** | **Year** | **Gen lab** | **N** |
| --- | --- | --- | --- | --- | --- | --- | --- | --- | --- |
| ARA | Aracaju* [1] | Brazil | South America | | -10.909151 | -37.074454 | 2010 | 0 | 24 |
| BEL | Belem [2] | Brazil | South America | | -1.455807 | -48.490164 | 2010 | 0 | 30 |
| BOA | Boa Vista [3] | Brazil | South America | | 2.851155 | -60.661693 | 2010 | 0 | 28 |
| CA08 | Cachoeiro* [4] | Brazil | South America | | -20.849368 | -41.113221 | 2008 | 2 | 23 |
| CA12 | Cachoeiro* [5] | Brazil | South America | | -20.849368 | -41.113221 | 2012 | 0.5 | 47 |
| CAGR | Campo Grande [6] | Brazil | South America | | -20.469765 | -54.620237 | 2012 | 0 | 52 |
| CAST | Castanhal [7] | Brazil | South America | | -1.298588 | -47.916298 | 2011 | 0 | 29 |
| FORT | Fortaleza [8] | Brazil | South America | | -3.731913 | -38.526747 | 2004 | 0 | 23 |
| FOZ | Foz do Iguacu [9] | Brazil | South America | | -25.516208 | -54.585431 | 2009 | 0 | 30 |
| GOI | Goiania* [10] | Brazil | South America | | -16.677715 | -49.267630 | 2009 | 0 | 23 |
| ITA | Itacoatiara [11] | Brazil | South America | | -3.135026 | -58.438539 | 2014 | 0 | 30 |
| JAC | Jacobina* [12] | Brazil | South America | | -11.185452 | -40.536079 | 2013 | 0 | 94 |
| MACP | Macapa [13] | Brazil | South America | | 0.047797 | -51.066719 | 2012 | 0 | 59 |
| MAC | Maceio * [14] | Brazil | South America | | -9.666252 | -35.735098 | 2009 | 1 | 24 |
| MAR | Maraba * [15] | Brazil | South America | | -5.369968 | -49.116927 | 2010 | 0 | 48 |
| CLA | Montes Claros [16] | Brazil | South America | | -16.728619 | -43.858233 | 2006 | 0 | 30 |
| MOS | Mossoro* [17] | Brazil | South America | | -5.188036 | -37.344134 | 2009 | 1 | 21 |
| NAT | Natal* [18] | Brazil | South America | | -5.794478 | -35.210955 | 2010 | 0 | 47 |
| IGU | Nova Iguaçu [19] | Brazil | South America | | -22.756130 | -43.460742 | 2012 | 0 | 61 |
| PAC | Pacaraima [20] | Brazil | South America | | 4.203112 | -60.858225 | 2011 | 0 | 30 |
| PAR | Parnaiba [21] | Brazil | South America | | -2.905483 | -41.773280 | 2005 | 0 | 28 |
| PARM | Parnamirim [22] | Brazil | South America | | -5.911555 | -35.272316 | 2012 | 0 | 55 |
| FER | Pau dos Ferros* [23] | Brazil | South America | | -6.102490 | -38.209222 | 2009 | 1 | 15 |
| BRA | Rio Branco [24] | Brazil | South America | | -9.975455 | -67.824780 | 2011 | 0 | 28 |
| RIO | Rio de Janeiro [25] | Brazil | South America | | -22.906837 | -43.172872 | 2014 | 0 | 33 |
| SANR | Santanrém [26] | Brazil | South America | | -2.450629 | -54.700923 | 2012 | 0 | 77 |
| SAN | Santos [27] | Brazil | South America | | -23.967632 | -46.329030 | 2014 | 0 | 30 |
| GON | Sao Goncalo* [28] | Brazil | South America | | -22.827099 | -43.054379 | 2009 | 2 | 20 |
| PRE | SJR Preto [29] | Brazil | South America | | -20.811806 | -49.376095 | 2014 | 0 | 29 |
| TOC | Tocantins [30] | Brazil | South America | -10.194631 | | -48.330092 | 2012 | 0 | 29 |
| TUC | Tucurui * [31] | Brazil | South America | | -3.769528 | -49.674109 | 2010 | 0 | 17 |
| PANC | Pance de Cali [32] | Colombia | South America | | 3.328340 | -76.638650 | 2013 | 0 | 24 |
| CAL | Paso de Comercio Cali [33] | Colombia | South America | | 3.492860 | -76.488845 | 2013 | 0 | 56 |
| BOL | Bolivar* [34] | Venezuela | South America | | 7.739104 | -72.449485 | 2004 | 2 | 48 |
| ZUL | Zulia* [35] | Venezuela | South America | | 9.969138 | -72.524875 | 2004 | 2 | 47 |
| HOU | Houston TX* [36] | USA | North America | | 29.760425 | -95.369801 | 2009 | 0 | 29 |
| KWES | Key West FL [37] | USA | North America | | 24.555060 | -81.779988 | 2013 | 0 | 52 |
| MIA | Miami FL* [38] | USA | North America | | 25.761682 | -80.191799 | 2010 | 0 | 47 |
| AMAC | Amacuzac Morelos [39] | Mexico | North America | | 18.600446 | -99.370080 | 2013 | 0 | 54 |
| COA | Coatzacoalcos* [40] | Mexico | North America | | 18.134471 | -94.458982 | 2008 | 0 | 50 |
| PIJ | Pijijiapan* [41] | Mexico | North America | | 15.685171 | -93.212254 | 2008 | 1 | 47 |
| TIJ | Tijuana [42] | Mexico | North America | | 32.514947 | -117.038250 | 2013 | 0 | 20 |
| CR | Costa Rica [43] | Republic of Costa Rica | Central America | | 9.748917 | -83.753428 | 2014 | 0 | 51 |
| DOM | Dominica* [44] | Commonwealth of Dominica | Caribbean | | 15.414944 | -61.370977 | 2009 | 0 | 48 |
| PAT | Patillas Puerto Rico [45] | Commonwealth of Puerto Rico | Caribbean | | 18.220833 | -66.590149 | 2014 | 0 | 54 |
| PR | Puerto Rico* [46] | Commonwealth of Puerto Rico | Caribbean | | 18.220833 | -66.590149 | 2012 | 0 | 54 |
| CAR | Carriacou [47] | Grenada | Caribbean | | 12.478589 | -61.449384 | 2015 | 0 | 29 |
| TRI | Trinidad [48] | Republic of Trinidad and Tobago | Caribbean | | 10.375720 | -61.233616 | 2014 | 0 | 51 |

* Populations used in the Monteiro et al [11] study.
