# Supplementary material for: Tracking the return of Aedes aegypti to Brazil, the major vector of the dengue, chikungunya and Zika viruses
Source: PLoS Negl Trop Dis. 2017 Jul 25;11(7):e0005653. doi: 10.1371/journal.pntd.0005653 (PMC5526527; doi:10.1371/journal.pntd.0005653)
Supplement: S2 Table — Prior and posterior distribution of parameters of Approximate Bayesian Computation (ABC) analysis, to test hypothesis of re-colonization of Brazil by Ae. aegypti, using DIYABC software [46]. Priors for splitting time are all uniform distributions and those for population size are log-uniform. Posterior probabilities for each scenario depicted in Fig 2 are shown with 95% confidence intervals. Error and posterior distributions of parameters (median with 90% confidence interval) are reported for the best-fit scenario (Scenario 1 in Fig 2). Summary statistics used to compare simulated and observed data sets were: mean number of alleles, mean genetic diversity [60], mean size variance, and Fst. Divergence times are measured in generations. (DOCX) [file pntd.0005653.s005.docx]

**Table S2.** **Parameters of Approximate Bayesian Computation (ABC) analysis.**

| **Parameter** | **Details** | **Prior** | **Posterior** |
| --- | --- | --- | --- |
| Colonization | Scenario 1 | N/A | 0.845[0.807, 0.833] |
| scenario | Scenario 2 | N/A | 0.123[0.000, 0.334] |
|  | Scenario 3 | N/A | 0.021[0.000, 0.225] |
|  | Scenario 4 | N/A | 0.006[0.000, 0.212] |
|  | Scenario 5 | N/A | 0.005[0.000, 0.211] |
| 1Effective | Venezuela | 100 – 500,000 | 274000[25400, 490000] |
| population | USA | 100 – 500,000 | 65200[34800, 124000] |
| size | Dominica | 100 – 500,000 | 770[100, 71400] |
|  | North Brazil | 100 – 500,000 | 5000[321, 236000] |
|  | South Brazil | 100 – 500,000 | 8280[228, 313000] |
| Split time | USA-Venezuela | 100-5,000 | 4210[2390, 4920] |
|  | Venezuela-North Brazil | 100-500 | 437[186, 584] |
|  | North Brazil-South Brazil | 100-500 | 305[113, 509] |
|  | South Brazil-Dominica | 100-500 | 125[29.8, 342] |
| Mutation | Microsatellite –Stepwise Mutation | 9x10-06 - 1x10-05 | 9.5x10-06[9.08x10-06, 1x10-05] |
| Model | Model (SMM) |  |  |
| Confidence | Type I error | N/A | 0.298 |
|  | (Simulated under scenario 1) |  |  |
|  | Type II error | N/A | 0.132 |
|  | (Simulated under scenario 2) |  |  |
|  | Type II error | N/A | 0.048 |
|  | (Simulated under scenario 3) |  |  |
|  | Type II error | N/A | 0.004 |
|  | (Simulated under scenario 4) |  |  |
|  | Type II error | N/A | 0.004 |
|  | (Simulated under scenario 5) |  |  |
