# Supplementary material for: Tracking the return of Aedes aegypti to Brazil, the major vector of the dengue, chikungunya and Zika viruses
Source: PLoS Negl Trop Dis. 2017 Jul 25;11(7):e0005653. doi: 10.1371/journal.pntd.0005653 (PMC5526527; doi:10.1371/journal.pntd.0005653)
Supplement: S3 Table — Weir & Cockerham's [61] FIS estimated values using the Genepop software [15] for all Ae. aegypti populations for each microsatellite locus. Values that remained significant (alpha = 0.05) after sequential Bonferroni correction are indicated by bold characters. Abbreviations: NA: non available. (DOCX) [file pntd.0005653.s006.docx]

**Table S3. *Aedes aegypti* Fis values per locus and per population.**

| **Population** | **AC1** | **AC2** | **AC4** | **AC5** | **CT2** | **AG1** | **AG2** | **AG5** | **A1** | **A9** | **B2** | **B3** |
| --- | --- | --- | --- | --- | --- | --- | --- | --- | --- | --- | --- | --- |
| Maraba [15] | 0.093 | 0.135 | 0.239 | -0.208 | 0.088 | 0.049 | 0.188 | 0.081 | 0.083 | **0.230** | 0.086 | -0.245 |
| Natal [18] | -0.021 | -0.073 | -0.108 | 0.030 | -0.046 | 0.080 | -0.038 | -0.052 | -0.154 | 0.205 | 0.084 | 0.058 |
| Aracaju [01] | -0.170 | 0.030 | -0.140 | 0.372 | -0.022 | -0.053 | 0.099 | -0.046 | -0.124 | 0.288 | -0.022 | -0.095 |
| Goiania [10] | 0.034 | 0.101 | -0.144 | **0.472** | 0.403 | -0.079 | 0.083 | -0.058 | -0.336 | 0.448 | -0.128 | 0.428 |
| Maceio [14] | -0.325 | -0.266 | -0.095 | 0.452 | -0.022 | 0.017 | -0.125 | 0.183 | -0.085 | 0.854 | -0.122 | 0.322 |
| Mossoro [17] | -0.104 | 0.391 | -0.226 | 0.123 | -0.053 | 0.062 | 0.060 | -0.212 | -0.226 | 0.437 | NA | -0.129 |
| Pau dos Ferros [23] | 0.031 | -0.026 | -0.189 | 0.527 | 0.048 | -0.020 | 0.245 | -0.202 | 0.333 | 0.676 | NA | 0.774 |
| Tucurui [31] | -0.393 | -0.161 | -0.778 | **0.411** | 0.033 | -0.378 | 0.090 | -0.025 | -0.122 | -0.237 | NA | 0.059 |
| Sao Goncalo [28] | -0.039 | 0.108 | -0.310 | -0.373 | 0.022 | 0.220 | 0.163 | -0.025 | 0.014 | **0.909** | -0.010 | -0.134 |
| Cachoeiro [04] | -0.058 | -0.204 | -0.128 | 0.083 | 0.057 | -0.123 | 0.283 | -0.257 | -0.068 | **0.677** | -0.048 | 0.033 |
| Cachoeiro [05] | -0.046 | 0.243 | 0.055 | 0.144 | 0.334 | -0.038 | -0.135 | 0.181 | -0.145 | 0.283 | -0.067 | 0.164 |
| Jacobina [12] | 0.177 | -0.124 | -0.017 | -0.053 | 0.145 | -0.132 | 0.045 | -0.114 | 0.074 | **0.423** | 0.073 | 0.269 |
| Rio de Janeiro [25] | 0.040 | -0.135 | -0.231 | 0.010 | 0.066 | -0.039 | 0.126 | -0.059 | 0.138 | **0.811** | 0.084 | 0.470 |
| SJR Preto [29] | -0.018 | 0.381 | **0.743** | **-0.136** | 0.336 | -0.099 | 0.036 | **0.093** | 0.382 | 0.507 | -0.098 | 0.414 |
| Santos [27] | -0.139 | 0.296 | 0.663 | 0.078 | 0.216 | -0.039 | 0.056 | 0.065 | -0.179 | **0.860** | 0.023 | -0.125 |
| Rio Branco [24] | 0.191 | 0.073 | **1.000** | -0.141 | -0.055 | -0.201 | -0.133 | 0.100 | 0.456 | 0.111 | 0.357 | 0.109 |
| Parnaiba [21] | 0.327 | 0.294 | **1.000** | **0.317** | -0.009 | 0.086 | 0.041 | 0.256 | 0.342 | **0.583** | -0.009 | -0.002 |
| Pacaraima [20] | 0.039 | -0.254 | NA | -0.080 | 0.015 | 0.140 | 0.153 | -0.027 | 0.069 | **0.817** | -0.177 | 0.236 |
| Montes Claros [16] | 0.123 | 0.275 | NA | -0.122 | -0.208 | -0.107 | 0.091 | -0.207 | -0.187 | 0.270 | -0.025 | 0.433 |
| Itacoatiara [11] | 0.221 | 0.085 | NA | -0.018 | -0.366 | 0.172 | **0.792** | -0.163 | -0.041 | **0.680** | 0.237 | -0.025 |
| Foz do Iguacu [09] | 0.049 | -0.187 | 0.370 | -0.018 | -0.069 | -0.132 | -0.135 | 0.052 | -0.159 | 0.059 | -0.186 | 0.064 |
| Fortaleza [08] | -0.138 | -0.130 | **1.000** | -0.004 | NA | -0.062 | 0.031 | -0.026 | -0.389 | **0.624** | NA | 0.436 |
| Castanhal [07] | -0.131 | -0.080 | **1.000** | -0.051 | 0.125 | 0.205 | 0.018 | -0.177 | 0.096 | **0.633** | -0.057 | -0.008 |
| Boa Vista [03] | -0.199 | 0.076 | NA | **0.421** | 0.393 | -0.067 | 0.286 | 0.164 | -0.153 | 0.487 | 0.198 | -0.054 |
| Belem [02] | 0.063 | -0.025 | **1.000** | **0.436** | 0.141 | 0.028 | 0.203 | 0.059 | -0.110 | 0.126 | -0.055 | 0.100 |
| Tocantins [30] | -0.044 | -0.176 | 0.173 | 0.010 | 0.213 | 0.110 | -0.087 | 0.162 | -0.064 | **0.776** | -0.217 | 0.149 |
| Parnamirim [22] | -0.001 | 0.168 | -0.111 | 0.070 | 0.114 | 0.046 | 0.004 | 0.114 | 0.210 | **0.613** | -0.103 | 0.290 |
| Macapa [13] | 0.047 | 0.084 | 0.295 | 0.007 | 0.094 | -0.140 | 0.125 | -0.019 | -0.005 | 0.320 | -0.094 | 0.041 |
| Campo Grande [06] | 0.094 | 0.020 | 0.394 | 0.125 | -0.041 | 0.033 | 0.163 | 0.008 | 0.256 | 0.074 | -0.010 | -0.097 |
| Nova Iguaçu [19] | 0.133 | 0.099 | 0.111 | 0.221 | 0.081 | -0.149 | 0.214 | 0.052 | -0.092 | **0.763** | -0.008 | 0.321 |
| Santarem [26] | -0.031 | -0.011 | 0.107 | 0.125 | 0.018 | 0.040 | 0.140 | -0.019 | 0.240 | -0.009 | 0.016 | -0.210 |
| Patillas Puerto Rico [45] | -0.023 | 0.103 | -0.006 | **-0.039** | 0.093 | -0.011 | 0.034 | -0.053 | -0.262 | 0.243 | 0.379 | -0.073 |
| Pance de Cali [32] | 0.033 | 0.019 | -0.190 | -0.007 | 0.266 | 0.282 | -0.090 | -0.150 | -0.165 | 0.438 | 0.195 | 0.115 |
| Paso de Comercio Cali [33] | **0.408** | -0.410 | -0.236 | -0.177 | 0.214 | -0.272 | **0.273** | **0.436** | -0.061 | 0.161 | NA | **0.166** |
| Tijuana [42] | -0.145 | **-0.374** | -0.148 | -0.338 | -0.016 | 0.206 | -0.154 | -0.031 | 0.020 | -0.024 | -0.188 | -0.016 |
| Key West [37] | -0.074 | 0.010 | 0.015 | 0.002 | -0.043 | 0.092 | -0.039 | -0.049 | -0.075 | 0.295 | 0.123 | -0.096 |
| Amacuzac [39] | -0.085 | 0.137 | -0.203 | **-0.180** | -0.029 | -0.037 | **0.004** | -0.022 | -0.128 | 0.008 | -0.159 | -0.067 |
| Costa Rica [43] | 0.249 | 0.124 | 0.051 | 0.049 | 0.157 | **-0.014** | 0.011 | 0.031 | 0.166 | **0.504** | 0.018 | -0.022 |
| Trinidad [48] | **0.320** | 0.003 | 0.123 | 0.186 | 0.388 | **0.164** | **-0.109** | **0.170** | 0.026 | 0.079 | **0.615** | -0.035 |
| Puerto Rico [46] | 0.126 | -0.184 | -0.063 | -0.119 | -0.245 | -0.111 | **-0.101** | **0.101** | **-0.101** | **-0.242** | NA | 0.234 |
| Carriacou [47] | **-1.000** | **-1.000** | -0.098 | -0.220 | -0.173 | -0.351 | -0.368 | **-0.443** | -0.385 | **0.216** | NA | **-0.592** |
| Dominica [44] | -0.013 | -0.176 | 0.793 | 0.026 | NA | -0.121 | 0.166 | -0.117 | -0.074 | **0.513** | NA | -0.203 |
| Pijijiapan [41] | 0.296 | 0.143 | 0.238 | 0.044 | NA | **0.501** | 0.130 | -0.031 | -0.074 | **0.319** | -0.011 | 0.044 |
| Coatzacoalcos [40] | -0.361 | 0.089 | -0.089 | -0.187 | NA | -0.258 | -0.290 | -0.077 | **-0.324** | -0.065 | NA | -0.111 |
| Bolivar [34] | **0.314** | 0.039 | -0.106 | 0.223 | -0.089 | -0.087 | 0.019 | 0.087 | 0.234 | 0.100 | -0.296 | -0.028 |
| Zulia [35] | -0.103 | -0.180 | 0.004 | **0.006** | -0.133 | -0.134 | 0.095 | 0.035 | 0.005 | -0.089 | -0.136 | 0.102 |
| Houston [36] | -0.203 | 0.743 | -0.122 | **-0.431** | -0.400 | 0.090 | -0.193 | **-0.291** | -0.280 | -0.106 | NA | -0.063 |
| Miami [38] | -0.057 | -0.172 | -0.398 | -0.114 | -0.058 | 0.078 | -0.166 | 0.054 | -0.137 | 0.264 | -0.122 | -0.004 |
