# Supplementary material for: Tracking the return of Aedes aegypti to Brazil, the major vector of the dengue, chikungunya and Zika viruses
Source: PLoS Negl Trop Dis. 2017 Jul 25;11(7):e0005653. doi: 10.1371/journal.pntd.0005653 (PMC5526527; doi:10.1371/journal.pntd.0005653)
Supplement: S4 Table — Null allele frequencies for each of the 12 microsatellite markers and the populations with at least one possible null allele at a given locus as estimated using the Microchecker software [20]. (DOCX) [file pntd.0005653.s007.docx]

**Table S4. Null allele frequencies.**

| **Locality [map code]** | **AC1** | **AC2** | **AC4** | **AC5** | **CT2** | **AG1** | **AG2** | **AG5** | **A1** | **A9** | **B2** | **B3** |
| --- | --- | --- | --- | --- | --- | --- | --- | --- | --- | --- | --- | --- |
| Belem [02] |  |  | 0.263 | 0.170 |  |  | 0.084 |  |  |  |  |  |
| Boa Vista [03] |  |  |  | 0.142 |  |  |  |  |  |  |  |  |
| Cachoeiro [04] |  |  |  |  |  |  | 0.084 |  |  | 0.226 |  |  |
| Cachoeiro [05] |  | 0.081 |  |  | 0.099 |  |  |  |  |  |  |  |
| Campo Grande [06] |  |  | 0.098 |  |  |  |  |  | 0.091 |  |  |  |
| Castanhal [07] |  |  | 0.247 |  |  |  |  |  |  | 0.240 |  |  |
| Fortaleza [08] |  |  | 0.312 |  |  |  |  |  |  | 0.188 |  |  |
| Goiania [10] |  |  |  | 0.201 |  |  |  |  |  | 0.143 |  | 0.143 |
| Itacoatiara [11] |  |  |  |  |  |  | 0.301 |  |  | 0.230 |  |  |
| Jacobina [12] | 0.058 |  |  |  |  |  |  |  |  | 0.166 |  | 0.065 |
| Macapa [13] |  |  |  |  |  |  |  |  |  | 0.117 |  |  |
| Maceio [14] |  |  |  | 0.099 |  |  |  |  |  | 0.317 |  |  |
| Maraba [15] |  |  |  |  |  |  | 0.074 |  |  |  |  |  |
| Montes Claros [16] |  |  |  |  |  |  |  |  |  | 0.098 |  | 0.162 |
| Nova Iguacu [19] |  |  |  |  |  |  | 0.080 |  |  | 0.288 |  | 0.082 |
| Pacaraima [20] |  |  |  |  |  |  |  |  |  | 0.280 |  |  |
| Parnaiba [21] |  |  | 0.161 | 0.129 |  |  |  |  | 0.096 | 0.224 |  |  |
| Parnamirim [22] |  |  |  |  |  |  |  |  |  | 0.228 |  | 0.081 |
| Pau dos Ferros [23] |  |  |  | 0.201 |  |  |  |  |  | 0.261 |  | 0.165 |
| Rio Branco [24] |  |  | 0.117 |  |  |  |  |  | 0.110 |  |  |  |
| Rio de Janeiro [25] |  |  |  |  |  |  |  |  |  | 0.308 |  | 0.115 |
| Santanrém[26] |  |  |  |  |  |  |  |  | 0.080 |  |  |  |
| Santos [27] |  |  | 0.057 |  |  |  |  |  |  | 0.296 |  |  |
| Sao Goncalo [28] |  |  |  |  |  |  |  |  |  | 0.319 |  |  |
| SJR Preto [29] |  | 0.131 | 0.084 |  |  |  |  |  | 0.132 | 0.174 |  | 0.102 |
| Tocantins [30] |  |  |  |  |  |  |  |  |  | 0.237 |  |  |
| Tucurui [31] |  |  |  | 0.154 |  |  |  |  |  |  |  |  |
| Pance de Cali [32] |  |  |  |  |  | 0.111 |  |  |  | 0.157 |  |  |
| Paso de Comercio, Cali [33] | 0.123 |  |  |  |  |  | 0.080 | 0.164 |  |  |  |  |
| Bolivar [34] | 0.120 |  |  | 0.091 |  |  |  |  |  |  |  |  |
| Houston [36] |  | 0.085 |  |  |  |  |  |  |  |  |  |  |
| Key West [37] |  |  |  |  |  |  |  |  |  | 0.109 |  |  |
| Miami [38] |  |  |  |  |  |  |  |  |  | 0.101 |  |  |
| Pijijiapan [41] | 0.103 |  |  |  |  | 0.200 |  |  |  | 0.122 |  |  |
| Costa Rica [43] | 0.098 |  |  |  |  |  |  |  |  | 0.197 |  |  |
| Dominica [44] |  |  | 0.055 |  |  |  | 0.084 |  |  | 0.184 |  |  |
| Patillas Puerto Rico [45] |  |  |  |  |  |  |  |  |  | 0.096 |  |  |
| Puerto Rico [46] |  |  |  |  |  |  |  |  |  |  |  | 0.075 |
| Trinidad [48] | 0.098 |  |  |  | 0.116 |  |  | 0.069 |  |  | 0.122 |  |
