# Supplementary material for: Tracking the return of Aedes aegypti to Brazil, the major vector of the dengue, chikungunya and Zika viruses
Source: PLoS Negl Trop Dis. 2017 Jul 25;11(7):e0005653. doi: 10.1371/journal.pntd.0005653 (PMC5526527; doi:10.1371/journal.pntd.0005653)
Supplement: S5 Table — Locus-by-locus test for Linkage Disequilibrium (LD) for all 48 populations under study as implemented in Genepop software [15]. Only cases that remained significant after Bonferroni correction (p<0.05) for multiple tests are presented. Numbers in brackets after the population name are as in S1 Table and Fig 1A. (DOCX) [file pntd.0005653.s008.docx]

**Table S5**. **Linkage Disequilibrium (LD) test.**

| Population [map code] | Pair-loci |
| --- | --- |
| Puerto Rico [46] | A1-A9 |
| Amacuzac [39] | A1-B3 |
| Coatzacoalcos [40] | A1-B3 |
| Paso de Comercio, Cali [33] | A1-B3 |
| Puerto Rico [46] | A1-B3 |
| Coatzacoalcos [40] | AC1-A1 |
| Paso de Comercio, Cali [33] | AC1-A1 |
| Puerto Rico [46] | AC1-A1 |
| Paso de Comercio, Cali [33] | AC1-A9 |
| Puerto Rico [46] | AC1-A9 |
| Amacuzac [39] | AC1-AC4 |
| Coatzacoalcos [40] | AC1-AC4 |
| Houston [36] | AC1-AC4 |
| Paso de Comercio, Cali [33] | AC1-AC5 |
| Paso de Comercio, Cali [33] | AC1-AG1 |
| Amacuzac [39] | AC1-AG2 |
| Paso de Comercio, Cali [33] | AC1-AG2 |
| Paso de Comercio, Cali [33] | AC1-AG5 |
| Amacuzac [39] | AC1-B3 |
| Coatzacoalcos [40] | AC1-B3 |
| Houston [36] | AC1-B3 |
| Paso de Comercio, Cali [33] | AC1-B3 |
| Paso de Comercio, Cali [33] | AC1-CT2 |
| Puerto Rico [46] | AC2-A1 |
| Puerto Rico [46] | AC2-A9 |
| Coatzacoalcos [40] | AC2-AC5 |
| Puerto Rico [46] | AC2-AC5 |
| Paso de Comercio, Cali [33] | AC2-AG1 |
| Puerto Rico [46] | AC2-AG1 |
| Tijuana [42] | AC2-AG1 |
| Puerto Rico [46] | AC2-AG2 |
| Houston [36] | AC4-A1 |
| Puerto Rico [46] | AC4-A1 |
| Amacuzac [39] | AC4-B3 |
| Coatzacoalcos [40] | AC4-B3 |
| Houston [36] | AC4-B3 |
| Paso de Comercio, Cali [33] | AC4-B3 |
| Tijuana [42] | AC4-B3 |
| Houston [36] | AC5-A1 |
| Key West [37] | AC5-A1 |
| Puerto Rico [46] | AC5-A9 |
| Aracaju [01] | AC5-AG1 |
| Puerto Rico [46] | AC5-AG1 |
| Paso de Comercio, Cali [33] | AC5-AG2 |
| Puerto Rico [46] | AC5-AG2 |
| Key West [37] | AC5-AG5 |
| Paso de Comercio, Cali [33] | AC5-AG5 |
| Natal [18] | AC5-B2 |
| Paso de Comercio, Cali [33] | AC5-B3 |
| Carriacou [47] | AC5-CT2 |
| Key West [37] | AC5-CT2 |
| Pance de Cali [32] | AC5-CT2 |
| Paso de Comercio, Cali [33] | AC5-CT2 |
| Puerto Rico [46] | AC5-CT2 |
| Tijuana [42] | AC5-CT2 |
| Key West [37] | AG1-AG5 |
| Paso de Comercio, Cali [33] | AG1-AG5 |
| Paso de Comercio, Cali [33] | AG1-B3 |
| Puerto Rico [46] | AG2-A1 |
| Puerto Rico [46] | AG2-A9 |
| Amacuzac [39] | AG2-AG5 |
| Paso de Comercio, Cali [33] | AG2-AG5 |
| Patillas, Puerto Rico [45] | AG2-AG5 |
| Puerto Rico [46] | AG2-AG5 |
| Trinidad [48] | AG2-AG5 |
| Amacuzac [39] | AG2-B2 |
| Tijuana [42] | AG2-B2 |
| Pance de Cali [32] | AG2-B3 |
| Paso de Comercio, Cali [33] | AG2-B3 |
| Puerto Rico [46] | AG2-B3 |
| Paso de Comercio, Cali [33] | AG5-A1 |
| Puerto Rico [46] | AG5-A1 |
| Puerto Rico [46] | AG5-A9 |
| Paso de Comercio, Cali [33] | AG5-B3 |
| Puerto Rico [46] | AG5-B3 |
| Trinidad [48] | CT2-A9 |
| Pance de Cali [32] | CT2-AG1 |
| Paso de Comercio, Cali [33] | CT2-AG1 |
| Trinidad [48] | CT2-AG2 |
| Paso de Comercio, Cali [33] | CT2-AG5 |
| Paso de Comercio, Cali [33] | CT2-B3 |
