# Supplementary material for: Tracking the return of Aedes aegypti to Brazil, the major vector of the dengue, chikungunya and Zika viruses
Source: PLoS Negl Trop Dis. 2017 Jul 25;11(7):e0005653. doi: 10.1371/journal.pntd.0005653 (PMC5526527; doi:10.1371/journal.pntd.0005653)
Supplement: S7 Table — Assignment of all Ae. aegypti samples to the genetic groups based on the Q-matrix retrieved from CLUMPP [26] for each STRUCTURE-defined group (in light gray Major Cluster1; Clusters 1A, 1B; sub-clusters 1A1, 1A2, 1A3, 1B1, 1B2 and in dark gray Major Cluster2; Clusters 2A, 2B; sub-clusters 2A1, 2A2, 2A3) and each DAPC-inferred group (Group1, Group2, Group3). Cases exhibiting admixed ancestry (0.50<Q value<0.62) based on the STRUCTURE analyses, are indicated by bold characters and Q values for all the structure groups are provided. In the last column the percentage (%) of correct assignment (alpha = 0.01) back to the reference population or defined STRUCTURE-cluster as estimated by the Geneclass2 [30] is presented. Population codes are as in S1 Table and Fig 1. Abbreviations: Pop. Q value: Q values retained for each population from CLUMPP which summarizes the Q values form the 10 independent STRUCTURE runs, Ind. Range: Range for the Q values retained for each individual mosquito, Pop: population, N: number of individuals. (PDF) [file pntd.0005653.s010.pdf]

Table S7. Summary of the Q values for STRUCTURE-defined groups, assignment to DAPC-groups and result from assignment tests for all populations and groups of the study.

			GENETIC STRUCTURE (STRUCTURE)									DAPC (ADEGENET)			Assignment (Geneclass2)
			Q values									Number of individuals			
			Preliminary		separate clusters analysis							DAPC-inferred group (Ncluster1/Ncluster2)			
	Population [code]	N	Pop. Q value	Ind. Range	1A/2A	1B/2B	1A1/2A1	1A2/2A2	1A3/2A3	1B1	1B2	Group1 (314/290)	Group2 (299/417)	Group3 (201/354)	% correct assignment
MAIN CLUSTER 1 (preliminary STRUCTURE analysis)	Natal [18]	47	0.93	0.53-0.99	0.81		0.76					26	15	6	23.40
	Parnamirim [22]	55	0.95	0.60-0.99	0.65		0.71					17	26	12	61.82
		102					*structure sub-cluster1A1*					*43*	*18*	*41*	*49.02*
	Aracaju [01]	24	0.86	0.24-0.99	0.84			0.86				15	6	3	66.67
	Fortaleza [08]	23	0.90	0.02-0.99	0.93			0.70				18	4	1	56.52
	Mossoro [17]	21	0.96	0.82-0.99	0.94			0.87				15	6	0	80.95
	Parnaiba [21]	28	0.77	0.08-0.99	0.66		**0.25**	**0.50**	**0.25**			14	6	8	50.00
	Pau dos Ferros [23]	15	0.71	0.07-0.98	0.79		**0.32**	**0.51**	**0.17**			5	6	4	80.00
	Santos [27]	30	**0.62**	**0.06-0.96**	**0.50**	**0.50**		0.86				14	2	14	83.33
		141					*structure sub-cluster1A2*					*81*	*30*	*30*	*94.33*
	Cachoeiro [04]	23	0.92	0.71-0.99	0.70				0.68			16	1	6	8.70
	Cachoeiro [05]	47	0.95	0.72-0.99	**0.50**	**0.50**			0.79			23	0	24	38.30
	Jacobina [12]	94	0.95	0.08-0.98	0.62		**0.43**	**0.03**	**0.54**			44	30	20	29.79
	Maceio [14]	24	0.85	0.27-0.98	0.78		**0.32**	**0.10**	**0.58**			13	6	5	45.83
	Nova Iguaçu [19]	61	0.94	0.55-0.99	0.70				0.67			33	9	19	29.95
	Rio de Janeiro [25]	33	0.96	0.65-0.99	0.81		**0.45**	**0.05**	**0.50**			17	11	5	24.24
	Sao Goncalo [28]	20	0.85	0.18-0.99	**0.51**	**0.49**			0.77			10	1	9	76.19
		302					*structure sub-cluster1A3*					*156*	*88*	*58*	*31.13*
	Campo Grande [6]	52	0.93	0.46-0.99		0.79				0.96		6	0	46	9.61
	Foz DoIguacu [9]	30	0.76	0.13-0.99		0.93				0.82		0	6	24	93.33
	Goiania [10]	23	0.81	0.28-0.98		0.79				0.97		10	1	9	65.20
	Montes Claros [16]	30	0.83	0.26-0.98		0.73				0.74		7	5	18	53.33
	Sao Jose Rio Preto [29]	29	0.94	0.74-0.99		0.80				0.96		7	1	21	57.72
	Tocantins [30]	29	**0.61**	**0.03-0.96**		0.74				0.88		6	1	22	44.83
		193					*structure sub-cluster1B1*					*32*	*148*	*13*	*84.46*
	Rio Branco [24]	28	0.77	0.04-0.98		0.88					0.89	0	23	5	89.29
	Dominica [44]	48	0.92	0.35-0.99		0.86					0.96	2	36	10	93.75
		76					*structure sub-cluster1B2*					*2*	*15*	*59*	*88.16*
MAIN CLUSTER 2 (preliminary STRUCTURE analysis)	Belem [02]	30	0.88	0.25-0.99	0.90		0.86					9	4	17	80.00
	Castanhal [7]	29	0.78	0.06-0.98	0.83		0.72					9	6	14	82.76
	Macapa [13]	59	0.89	0.09-0.99	0.91		0.84					21	10	28	33.90
	Maraba [15]	48	0.88	0.30-0.99	0.94		0.9					17	5	26	20.83
	Santanrém [26]	77	0.78	0.07-0.99	0.94		0.73					66	5	6	64.94
	Tucurui [31]	17	0.68	0.21-0.97	0.80		0.93					11	0	6	29.42
		260					*structure sub-cluster2A1*					*133*	*30*	*97*	*81.54*
	Pance Cali [32]	24	0.74	0.04-0.98	0.68				0.76			0	12	12	91.67
	Paso Cali [33]	56	0.95	0.87-0.99	0.79				0.95			0	13	43	60.71
	Zulia [35]	47	0.90	0.45-0.99	0.63				0.74			2	4	41	55.32
		127					*structure sub-cluster2A2*					*2*	*29*	*96*	*61.42*
	Boa Vista [03]	28	0.76	0.04-0.99	0.94			0.95				21	2	5	60.71
	Itacoatiara [11]	30	**0.56**	**0.02-0.98**	0.86			0.79				15	5	10	60.00
	Pacaraima [20]	30	0.84	0.28-0.99	0.95			0.94				7	1	22	73.33
	Bolivar [34]	48	0.90	0.20-0.99	0.89			0.83				12	4	32	87.50
	Trinidad [48]	51	0.96	0.32-0.99	0.86			0.75				17	4	30	78.43
		187					*structure sub-cluster2A3*					*72*	*16*	*99*	*77.00*
	Houston [36]	27	0.94	0.69-0.98		0.95						0	28	1	82.76
	Key West [37]	52	0.95	0.78-0.99		0.92						5	37	10	48.08
	Miami [38]	47	0.95	0.52-0.99		0.87						1	36	10	87.23
	Amacuzac [39]	54	0.93	0.59-0.99		0.92						0	54	0	74.07
	Coatzacoalcos [40]	50	0.91	0.57-0.99		0.88						50	0	0	86.00
	Pijijiapan [41]	47	0.97	0.89-0.99		0.96						13	34	0	72.34
	Tijuana [42]	20	0.98	0.95-0.99		0.85						0	16	4	85.00
	Costa Rica [43]	51	0.76	0.04-0.99		0.95						7	43	1	84.31
	Patillas Puerto Rico [45]	54	0.79	0.14-0.98		0.64						4	31	19	81.48
	Puerto Rico [46]	54	0.85	0.11-0.98		0.75						3	34	17	42.59
	Carriacou [47]	29	0.94	0.72-0.99		0.96						0	29	0	100.00
		485					*structure sub-cluster2B*					*83*	*342*	*62*	*98.56*
