# Supplementary material for: Tracking the return of Aedes aegypti to Brazil, the major vector of the dengue, chikungunya and Zika viruses
Source: PLoS Negl Trop Dis. 2017 Jul 25;11(7):e0005653. doi: 10.1371/journal.pntd.0005653 (PMC5526527; doi:10.1371/journal.pntd.0005653)
Supplement: S8 Table — Fst values as estimated by FreeNA (below diagonal) and Number of private allele richness (Np) (on diagonal in bold type) as estimated by HPrare (assuming 100 genes) between Ae. aegypti populations pooled in geographic groups (1–8) or in STRUCTURE defined clusters (9–10). All Fst values were significant in Arlequin estimates (p<0.05). (DOCX) [file pntd.0005653.s011.docx]

**Table S8. Fst values and private alleles between *Ae. aegypti* populations pooled in geographic groups or in STRUCTURE defined clusters.**

|  | 1 | 2 | 3 | 4 | 5 | 6 | 7 | 8 | 9 | 10 |
| --- | --- | --- | --- | --- | --- | --- | --- | --- | --- | --- |
| 1. Caribbean* | **0.24** |  |  |  |  |  |  |  |  |  |
| 2. USA | 0.085 | **0.15** |  |  |  |  |  |  |  |  |
| 3. Trinidad | 0.110 | 0.168 | **0.09** |  |  |  |  |  |  |  |
| 4. Dominica | 0.144 | 0.140 | 0.275 | **0.14** |  |  |  |  |  |  |
| 5. Venezuela | 0.106 | 0.079 | 0.141 | 0.195 | **0.05** |  |  |  |  |  |
| 6. Colombia | 0.148 | 0.137 | 0.208 | 0.242 | 0.100 | **0.12** |  |  |  |  |
| 7. Mexico | 0.077 | 0.057 | 0.164 | 0.117 | 0.123 | 0.187 | **0.13** |  |  |  |
| 8. Costa Rica | 0.082 | 0.083 | 0.207 | 0.182 | 0.130 | 0.192 | 0.091 | **0.04** |  |  |
| 9. Cluster1_Brazil** | 0.077 | 0.107 | 0.180 | 0.127 | 0.117 | 0.142 | 0.089 | 0.105 | **0.54** |  |
| 10. Cluster2_Brazil** | 0.060 | 0.084 | 0.107 | 0.152 | 0.065 | 0.137 | 0.084 | 0.124 | 0.062 | **0.21** |

* Excluding Trinidad and Dominica, analyzed separately.

**Only Brazilian populations included.
