# Supplementary material for: Tracking the return of Aedes aegypti to Brazil, the major vector of the dengue, chikungunya and Zika viruses
Source: PLoS Negl Trop Dis. 2017 Jul 25;11(7):e0005653. doi: 10.1371/journal.pntd.0005653 (PMC5526527; doi:10.1371/journal.pntd.0005653)
Supplement: S9 Table — F scores and p values of the One-way ANOVA applied for the Heterozygosity, Private alleles, number of alleles and Allelic richness. In cases of statistical significance (p<0.05) post-hoc Tukey and Bonferroni tests were applied. Statistical significant cases (p<0.05) are indicated by bold characters. (DOCX) [file pntd.0005653.s012.docx]

**Table S9. ANOVA analyses of populations within Cluster 1 or Cluster 2 and the non-eradicated areas.**

|  | ANOVA | | | post-hoc test | |
| --- | --- | --- | --- | --- | --- |
| Groups | Parameter | F | p value | Tukey HSD | Bonferroni |
| 1. Cluster1_eradicated 2. Cluster2_eradicated 3. non eradicated areas | Heterozygosity | 12.516 | **<0.001** | **1-3; <0.001** | **1-3; <0.001** |
|  |  |  |  | **2-3; 0.016** | **2-3; 0.017** |
|  |  |  |  | 1-2; 0.339 | 1-2; 0.488 |
|  | Private Alleles | 0.173 | 0.914 |  |  |
|  | Alleles | 2.249 | 0.095 |  |  |
|  | Allelic richness | 1.160 | 0.32 |  |  |
