# Supplementary material for: Tracking the return of Aedes aegypti to Brazil, the major vector of the dengue, chikungunya and Zika viruses
Source: PLoS Negl Trop Dis. 2017 Jul 25;11(7):e0005653. doi: 10.1371/journal.pntd.0005653 (PMC5526527; doi:10.1371/journal.pntd.0005653)
Supplement: S10 Table — The percentage of within groups variation as estimated by AMOVA in the GenAlex software for different levels of grouping for the two Structure-defined Clusters 1 and 2 as in Fig 3 and Fig 4, respectively. (DOCX) [file pntd.0005653.s013.docx]

**Table S10. Within groups variation (AMOVA).**

| **Level** | **Cluster1** | **Cluster2** |
| --- | --- | --- |
| Populations | 81 | 71 |
| Structure K=2 | 94 | 92 |
| Structure K=5 | 87 | NA |
| Structure K=6 | NA | 83 |
