# Supplementary material for: Tracking the return of Aedes aegypti to Brazil, the major vector of the dengue, chikungunya and Zika viruses
Source: PLoS Negl Trop Dis. 2017 Jul 25;11(7):e0005653. doi: 10.1371/journal.pntd.0005653 (PMC5526527; doi:10.1371/journal.pntd.0005653)
Supplement: S11 Table — First generation migrants (Nmigrants) for Ae. aegypti populations between the major geographic regions of the study area and genetically defined clusters, as estimated by Geneclass2 [30] considering two levels of significance (p<0.05 and <0.01). The most possible source of migrants is also presented. At the lower part of the Table the assignment of individuals to groups according to ONCOR is presented. Individuals assigned to “their own” group are underlined. (DOCX) [file pntd.0005653.s014.docx]

**Table S11**. **First generation migrants estimated by Geneclass2 and assignment test results estimated by ONCOR.**

|  |  | **Nmigrants (p=0.05/p=0.01)** | **Most possible source of migrants based on the likelihood values of "L=L_home/L_max" as applied in Geneclass2 (Nmigrants at p=0.05/Nmigrants at p=0.01)** | | | | | | | | | |
| --- | --- | --- | --- | --- | --- | --- | --- | --- | --- | --- | --- | --- |
| **Group** | **N** |  | **1** | **2** | **3** | **4** | **5** | **6** | **7** | **8** | **9** | **10** |
| **1. Caribbean*** | 133 | 9/2 | 124/131 | 1/0 | 1/0 |  | 3/0 | 1/1 | 2/1 |  | 1/0 |  |
| **2. USA** | 123 | 11/4 | 2/1 | 112/119 |  |  | 3/2 |  |  |  | 4/1 | 2/0 |
| **3. Trinidad** | 50 | 5/1 | 4/0 |  | 45/49 |  |  |  | 1/1 |  |  |  |
| **4. Dominica** | 48 | 0/0 |  |  |  | 48/48 |  |  |  |  |  |  |
| **5. Venezuela** | 95 | 6/1 |  | 1/0 |  |  | 89/94 |  | 2/1 | 3/0 |  |  |
| **6. Cluster1_Brazil** | 705 | 45/11 | 4/1 | 2/0 |  | 5/1 | 3/1 | 666/694 | 23/4 | 1/0 | 3/1 | 4/2 |
| **7. Cluster2_Brazil** | 321 | 27/8 | 1/0 | 2/0 | 1/0 |  | 15/5 | 6/2 | 294/313 | 1/1 |  | 1/0 |
| **8. Colombia** | 80 | 7/1 | 2/0 |  |  |  | 3/0 | 2/1 |  | 72/79 |  |  |
| **9. Mexico** | 170 | 14/9 | 3/3 | 2/2 |  | 1/1 | 4/1 |  | 2/1 |  | 156/161 | 2/0 |
| **10.Costa Rica** | 46 | 5/1 | 1/1 | 4/0 |  |  |  |  |  |  |  | 41/45 |
| **Group** | **N** | **Largest misidentification (% to group)** | **Assignment of individuals to groups according to ONCOR software** | | | | | | | | | |
| **1. Caribbean*** | 133 | 3.8 % to Venezuela | 115 | 3 | 2 |  | 5 | 1 | 4 |  | 3 |  |
| **2. USA** | 123 | 4.1 % to Venezuela | 2 | 108 |  |  | 5 |  | 1 |  | 5 | 2 |
| **3. Trinidad** | 50 | 8.0 % to Caribbean | 4 |  | 45 |  |  |  | 1 |  |  |  |
| **4. Dominica** | 48 | 0.0 % |  |  |  | 48 |  |  |  |  |  |  |
| **5. Venezuela** | 95 | 4.2 % to Cluster2 |  | 3 |  |  | 88 |  | 4 |  |  |  |
| **6. Cluster1_Brazil** | 705 | 5.7 % to Cluster2 | 9 | 10 |  | 9 | 10 | 607 | 40 | 4 | 7 | 9 |
| **7. Cluster2_Brazil** | 321 | 7.5 % to Venezuela | 2 | 6 | 5 |  | 24 | 15 | 263 | 4 | 1 | 1 |
| **8. Colombia** | 80 | 3.8 % to Venezuela | 2 |  |  |  | 3 | 2 |  | 73 |  |  |
| **9. Mexico** | 170 | 2.9 % to Venezuela | 3 | 3 |  | 1 | 5 |  | 2 |  | 154 | 2 |
| **10.Costa Rica** | 46 | 8.7 % to USA | 1 | 4 |  |  |  |  |  |  |  | 41 |

* includes Carriacou and Puerto Rico.
