# Supplementary material for: Tracking the return of Aedes aegypti to Brazil, the major vector of the dengue, chikungunya and Zika viruses
Source: PLoS Negl Trop Dis. 2017 Jul 25;11(7):e0005653. doi: 10.1371/journal.pntd.0005653 (PMC5526527; doi:10.1371/journal.pntd.0005653)
Supplement: S12 Table — Results of the bottleneck tests for populations for which at least one method indicated significance population reductions. Results from both Wilcoxon test (TPM and SMM model) and the mode shift in allele frequency distributions are presented, as estimated by BOTTLENECK [34]. Significant values (p<0.05) are indicated with bold characters. (DOCX) [file pntd.0005653.s015.docx]

**Table S12**. **Evidence for Bottleneck**.

| **Population** | **p_W_TPM** | **p_W_SMM** | **mode shift** |
| --- | --- | --- | --- |
| Belem, Brazil | **0.005** | 0.455 | L-shaped |
| Castanhal, Brazil | **0.046** | 0.259 | L-shaped |
| Macapa, Brazil | **0.002** | 0.259 | L-shaped |
| Maraba, Brazil | **0.026** | 0.338 | L-shaped |
| Montes Claros, Brazil | **<0.001** | 0.051 | L-shaped |
| Pacaraima, Brazil | **0.006** | 0.415 | L-shaped |
| Pau dos Ferros, Brazil | **0.027** | 0.206 | L-shaped |
| Santanrem, Brazil | **0.032** | 0.661 | L-shaped |
| Carriacou, Caribbean | **<0.001** | **<0.001** | **shifted mode** |
| Dominica, Caribbean | **0.042** | 0.423 | L-shaped |
| Puerto Rico2012, Caribbean | **0.002** | 0.117 | L-shaped |
| Puerto Rico2014, Caribbean | **0.001** | 0.103 | **shifted mode** |
| Trinidad, Caribbean | **<0.001** | 0.339 | L-shaped |
| Pance Cali, Colombia | **0.002** | 0.260 | **shifted mode** |
| Paso Cali, Colombia | **0.032** | 0.133 | L-shaped |
| Pijijapan, Mexico | **0.051** | 0.483 | L-shaped |
| Tijuana, Mexico | **<0.001** | **<0.001** | **shifted mode** |
| Costa Rica, Rica | **0.021** | 0.065 | L-shaped |
| Key West, USA | **0.032** | 0.212 | L-shaped |
| Miami, USA | **0.002** | 0.259 | L-shaped |
| Zulia, Venezuela | **0.004** | 0.425 | L-shaped |
